# Supplementary material for: Deep generative model embedding of single-cell RNA-Seq profiles on hyperspheres and hyperbolic spaces
Source: Nat Commun. 2021 May 5;12:2554. doi: 10.1038/s41467-021-22851-4 (PMC8099904; doi:10.1038/s41467-021-22851-4)
Supplement: Supplementary file 1 — Supplementary Information [file 41467_2021_22851_MOESM1_ESM.pdf]

Supplementary Figures for

Deep generative model embedding of single-cell RNA-Seq profiles on hyperspheres and hyperbolic spaces

Jiarui Ding[1*] and Aviv Regev[1,2,3*]

[1]Klarman Cell Observatory, Broad Institute of MIT and Harvard, Cambridge MA 02142

[2]Howard Hughes Medical Institute, Koch Institute of Integrative Cancer Research, Department of Biology, Massachusetts Institute of Technology, Cambridge MA 02140

[3]Current address: Genentech, 1 DNA Way, South San Francisco, CA, 94080

*Correspondence: jding@broadinstitute.org (J.D.), aregev@broadinstitute.org (A.R.)

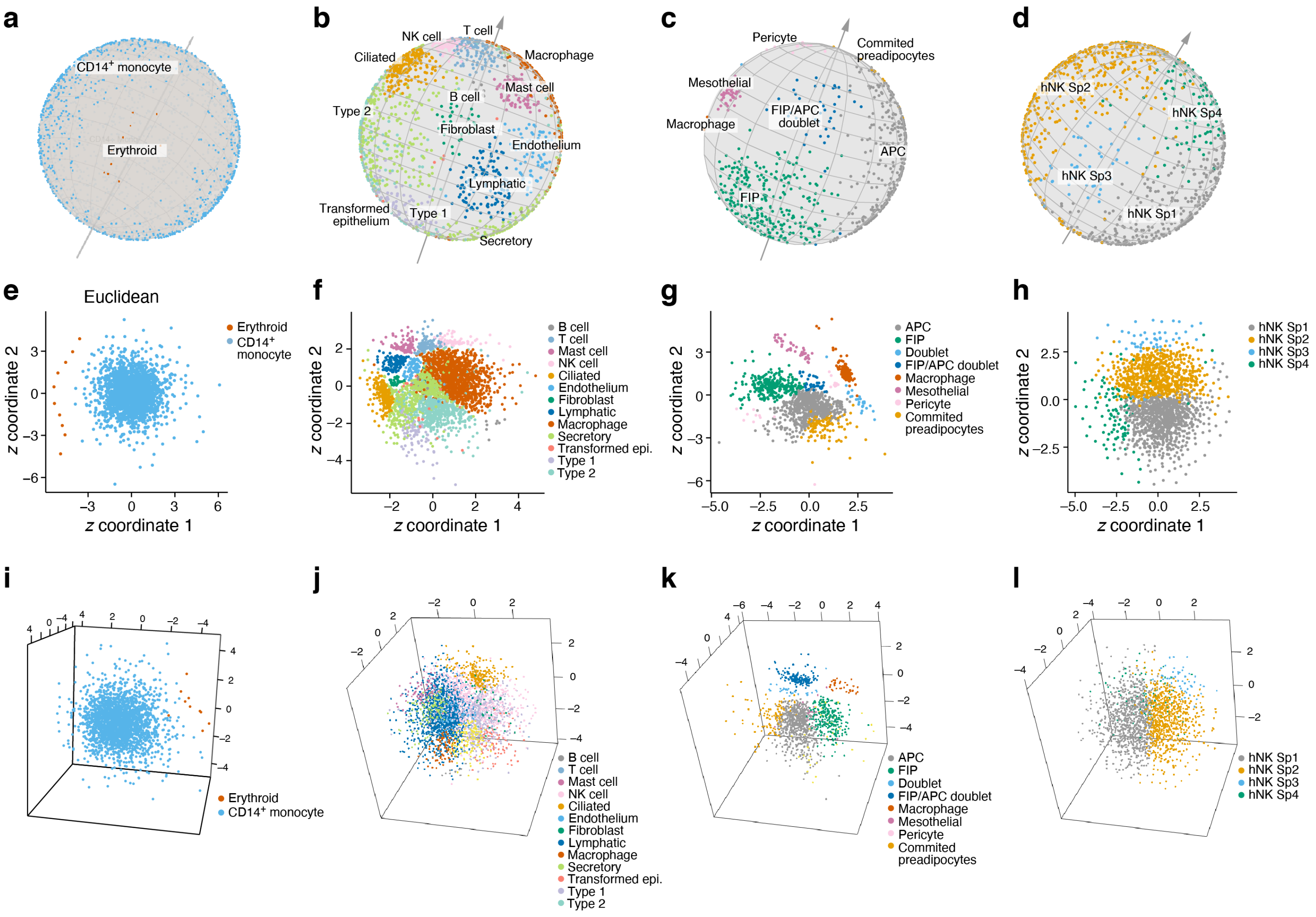

Supplementary Figure 1. The cell-crowding problem by embedding cells in the Euclidean space. ScPhere embeddings on the surface of unit spheres (a–d), 2D Euclidean space (e–h), or 3D Euclidean space (i–l) of 2,293 CD14^{+} monocytes and 10 erythroid cells (a, e, i), 3,314 human lung cells (b, f, j), 1,378 mouse white adipose tissue stromal cells (c, g, k), and 1,755 human splenic natural killer cells (d, h, l). Each dot represents a cell and is color coded by their annotated cell type.

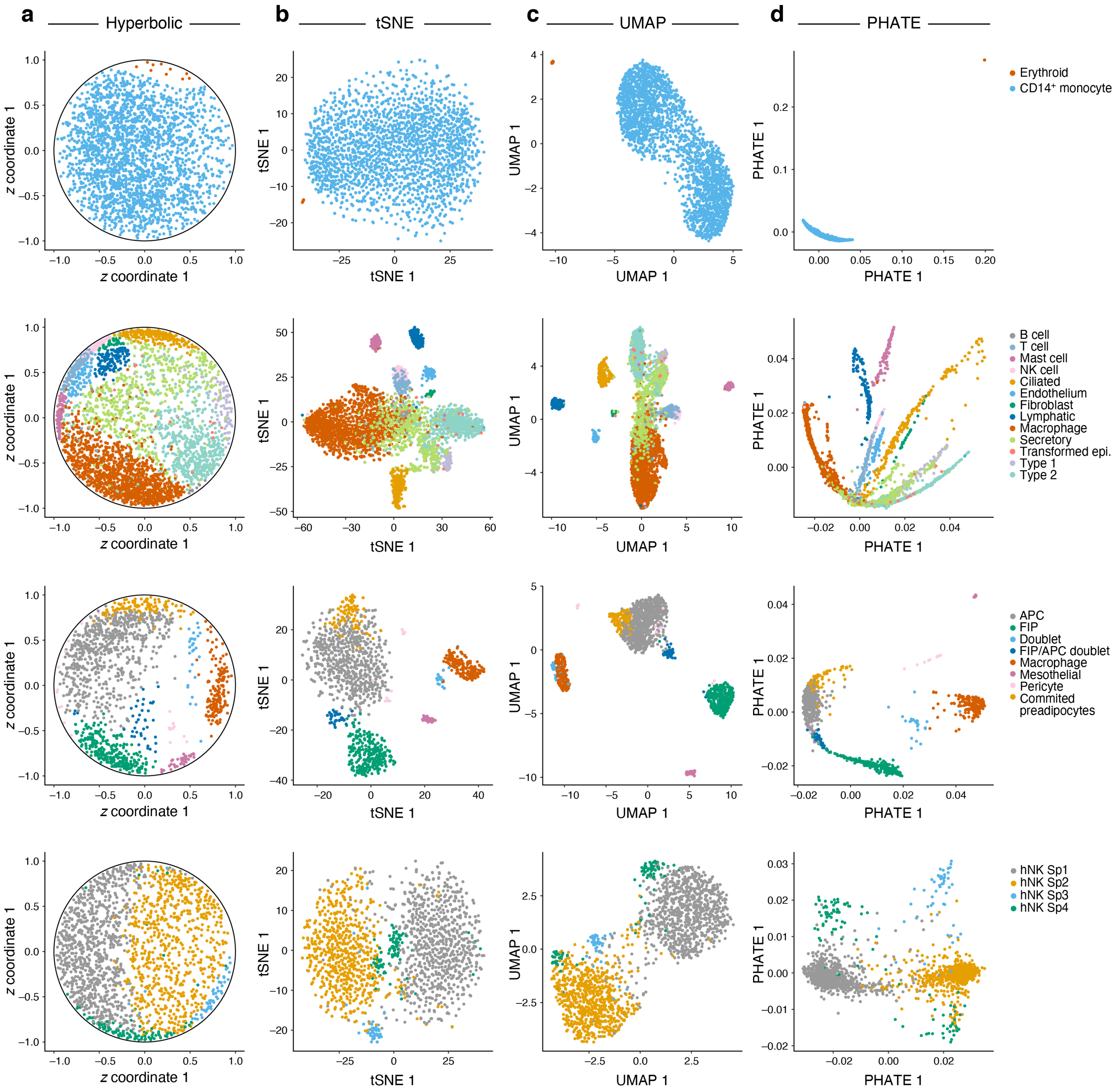

Supplementary Figure 2. Comparison of visualizations with scPhere with hyperbolic latent spaces, *t*-SNE, UMAP, and PHATE on smaller data sets. Visualizations with scPhere (with hyperbolic latent spaces) (a), 2D *t*-SNE (b), 2D UMAP (c), or 2D PHATE (d) of from top: 2,293 *CD14*$^{+}$ monocytes and 10 erythroid cells; 3,314 human lung cells; 1,378 mouse white adipose tissue stromal cells; and 1,755 human splenic natural killer cells. Each dot represents a cell and is color coded by their annotated cell type.

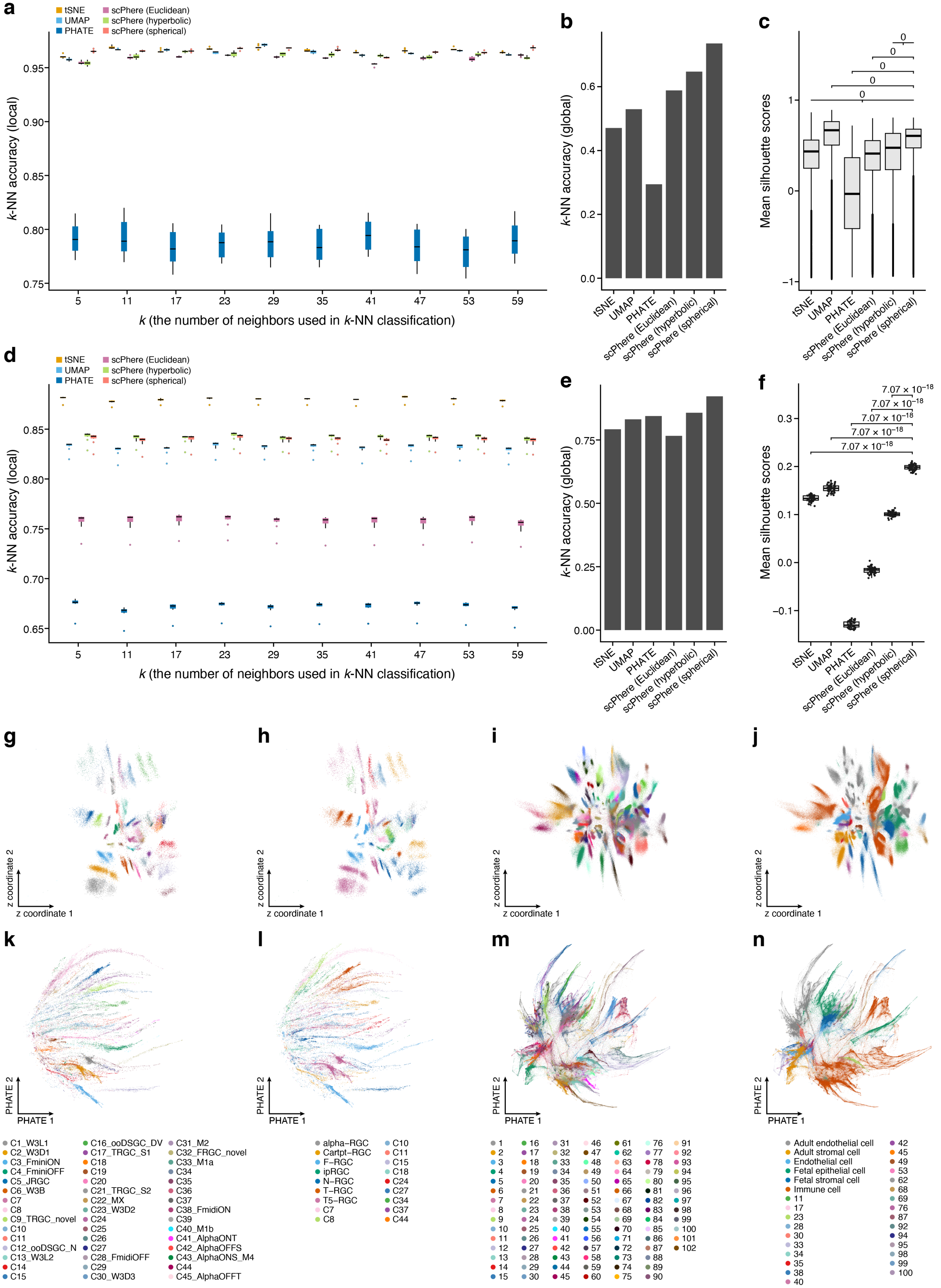
a
tSNE
UMAP
PHATE
scPhere (Euclidean)
scPhere (hyperbolic)
scPhere (spherical)
k-NN accuracy (local)
k (the number of neighbors used in k-NN classification)
b
k-NN accuracy (global)
c
Mean silhouette scores
d
e
f
7.07 × 10−18
g
h
i
j
z coordinate 1
z coordinate 2
k
l
m
n
PHATE 1
PHATE 2
C1_W3L1
C2_W3D1
C3_FminiON
C4_FminiOFF
C5_JRGC
C6_W3B
C7
C8
C9_TRGC_novel
C10
C11
C12_ooDSGC_N
C13_W3L2
C14
C15
C16_ooDSGC_DV
C17_TRGC_S1
C18
C19
C20
C21_TRGC_S2
C22_MX
C23_W3D2
C24
C25
C26
C27
C28_FmidiOFF
C29
C30_W3D3
C31_M2
C32_FRGC_novel
C33_M1a
C34
C35
C36
C37
C38_FmidiON
C39
C40_M1b
C41_AlphaONT
C42_AlphaOFFS
C43_AlphaONS_M4
C44
C45_AlphaOFFT
alpha−RGC
Cartpt−RGC
F−RGC
ipRGC
N−RGC
T−RGC
T5−RGC
Adult endothelial cell
Adult stromal cell
Endothelial cell
Fetal epithelial cell
Fetal stromal cell
Immune cell

Supplementary Figure 3. ScPhere outperforms other methods in preserving global and local structures and in addressing cell-crowding in visualizations. (a–f). Benchmarking for preservation of local structure, hierarchical global structure, and addressing cell-crowding in the RGC and HCL data sets. Local 10-fold *k*-NN accuracy (*y*-axis, a, d), global *k*-NN accuracy (b, e) and silhouette scores (c, f) across methods (*y*-axis) for the RGC (a–c) and HCL (d–f) data sets. Global accuracies were obtained from a leave-one-out cross-validation analysis on the condensed data sets where each point is a cluster center, and the cluster labels were the major cell types (or groups). For silhouette scores, we performed 50 repeated runs for the HCL data set, each a random sample of 20,000 cells. Boxplots denote the medians and the interquartile ranges (IQRs). Whiskers: lowest datum still within 1.5 IQR of the lower quartile and the highest datum still within 1.5 IQR of the upper quartile. *P*-values: two-sided Mann–Whitney *U* test adjusted *p*-values (FDR < 0.0001), (c) $n = 35{,}699$ cells and (f) $n = 50$ replicates. The adjusted *p*-values in (c) are smaller than the smallest number the *R* software can represent. g–n: ScPhere (in Euclidean latent space) (g–j) and PHATE (k–n) embedding of 35,699 RGCs (g, h, k, l) and 599,926 HCL cell profiles (i, j, m, n) colored by either cell type (g, i, k, m), or major cell groups (h, j, l, n).

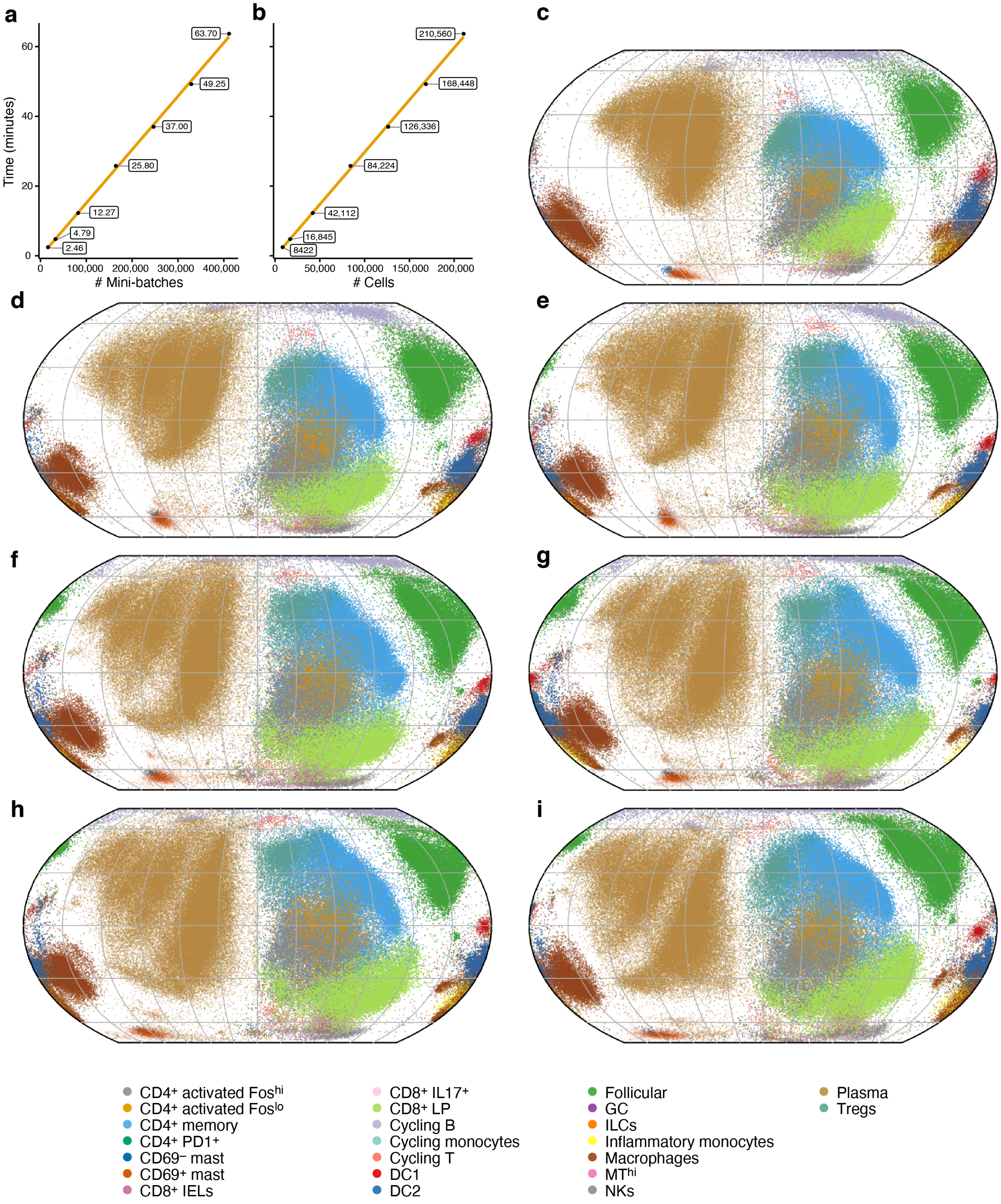
a
b
c
d
e
f
g
h
i
Time (minutes)
Mini-batches
Cells
63.70
49.25
37.00
25.80
12.27
4.79
2.46
210,560
168,448
126,336
84,224
42,112
16,845
8422
CD4+ activated Fos hi
CD4+ activated Fos lo
CD4+ memory
CD4+ PD1+
CD69− mast
CD69+ mast
CD8+ IELs
CD8+ IL17+
CD8+ LP
Cycling B
Cycling monocytes
Cycling T
DC1
DC2
Follicular
GC
ILCs
Inflammatory monocytes
Macrophages
MT hi
NKs
Plasma
Tregs

Supplementary Figure 4. ScPhere scales linearly with the number of mini-batches in training and the number of input cells. (a, b) Linear scaling with number of mini-batches or cells. ScPhere run time (*y*-axis) when using different numbers of mini-batches (*x*-axis, a; with 16,450, 32,900, 82,250, 164,500, 246,750, 329,000, and 411,250 mini-batches) with all 210,614 IBD immune cells, or the estimated equivalent number of cells (*x*-axis, b, assuming 128 mini-batches and 250 training epochs). (c–i) Quality of embedding is enhanced by extent of training. ScPhere embedding of the 210,614 IBD immune cells colored by subset (color legend) when we just trained the model for 10 epochs (16,450 min-batches; 2.45 minutes, c), and for each of the increasing larger number of mini-batches indicated in panel a (d–i).

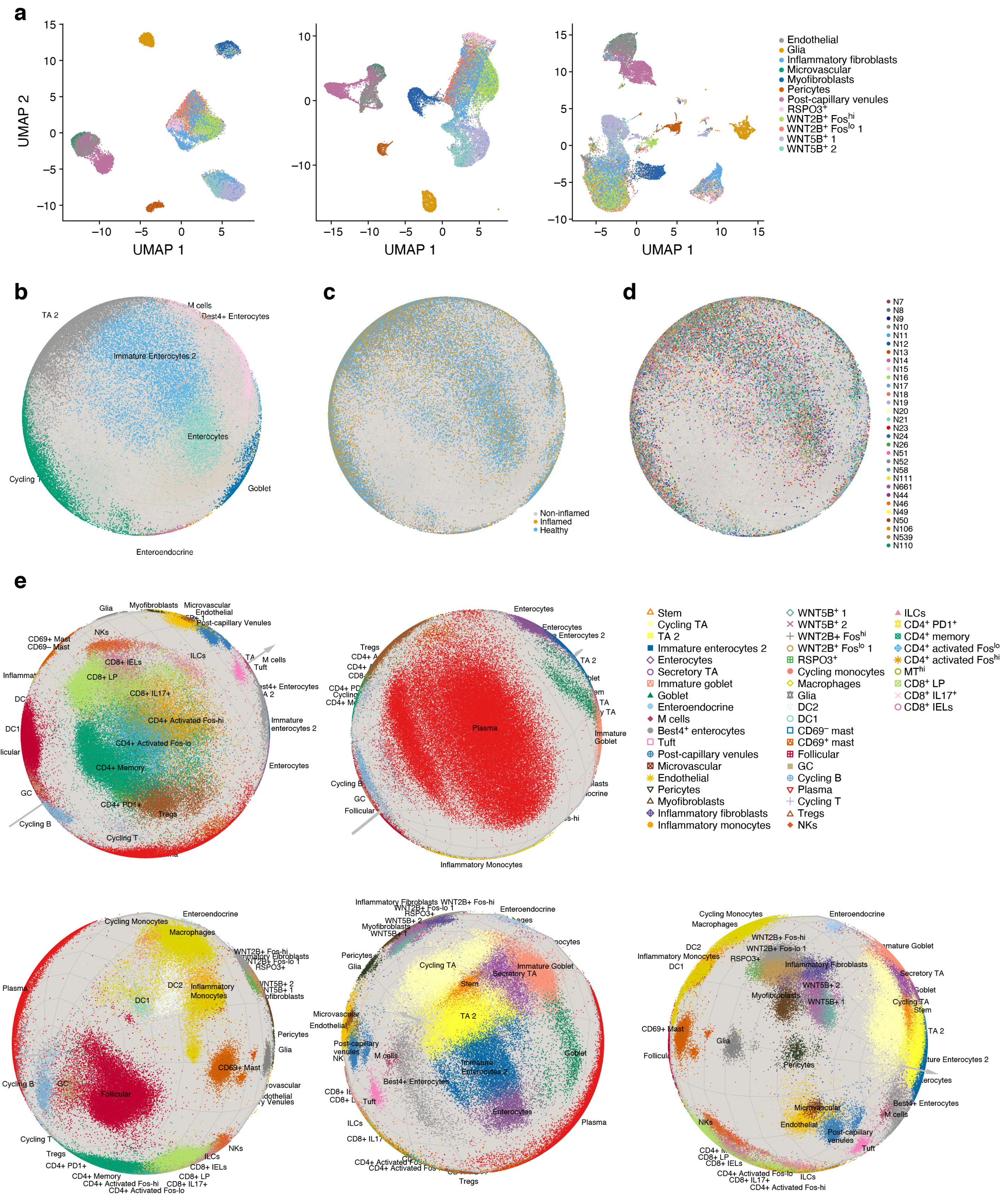

a
UMAP 2
UMAP 1
Endothelial
Glia
Inflammatory fibroblasts
Microvascular
Myofibroblasts
Pericytes
Post-capillary venules
RSPO3+
WNT2B+ Fos-hi
WNT2B+ Fos-lo 1
WNT5B+ 1
WNT5B+ 2
b
c
d
Non-inflamed
Inflamed
Healthy
N7
N8
N9
N10
N11
N12
N13
N14
N15
N16
N17
N18
N19
N20
N21
N23
N24
N26
N51
N52
N58
N111
N661
N44
N46
N49
N50
N106
N539
N110
e
Stem
Cycling TA
TA 2
Immature enterocytes 2
Enterocytes
Secretory TA
Immature goblet
Goblet
Enteroendocrine
M cells
Best4+ enterocytes
Tuft
Post-capillary venules
Microvascular
Endothelial
Pericytes
Myofibroblasts
Inflammatory fibroblasts
Inflammatory monocytes
WNT5B+ 1
WNT5B+ 2
WNT2B+ Fos-hi
WNT2B+ Fos-lo 1
RSPO3+
Cycling monocytes
Macrophages
Glia
DC2
DC1
CD69− mast
CD69+ mast
Follicular
GC
Cycling B
Plasma
Cycling T
Tregs
NKs
ILCs
CD4+ PD1+
CD4+ memory
CD4+ activated Fos-lo
CD4+ activated Fos-hi
MT-hi
CD8+ LP
CD8+ IL17+
CD8+ IELs

Supplementary Figure 5. ScPhere outperforms conventional approaches in analyzing and visualizing data sets with multi-level batch effects and complex structures. (a) Comparison to other batch correction methods. UMAP embedding of stromal cell scRNA-seq profiles, color coded by cell subset, when using 20 batch-corrected components from Harmony (left), Seurat3 CCA (middle), and LIGER (right). (b–d) ScPhere models address complex batch effects. ScPhere embedding of UC epithelial cells, taking patient, disease status, and anatomical region as the batch vector. Cells (dots) are colored by type (b), disease status (c), or patient (d). (e) ScPhere successfully embeds an entire colon mucosal data set. Different perspective of a sphere embedding (from Fig. 2n) of all epithelial, stromal, and immune cells, colored and labeled by type.

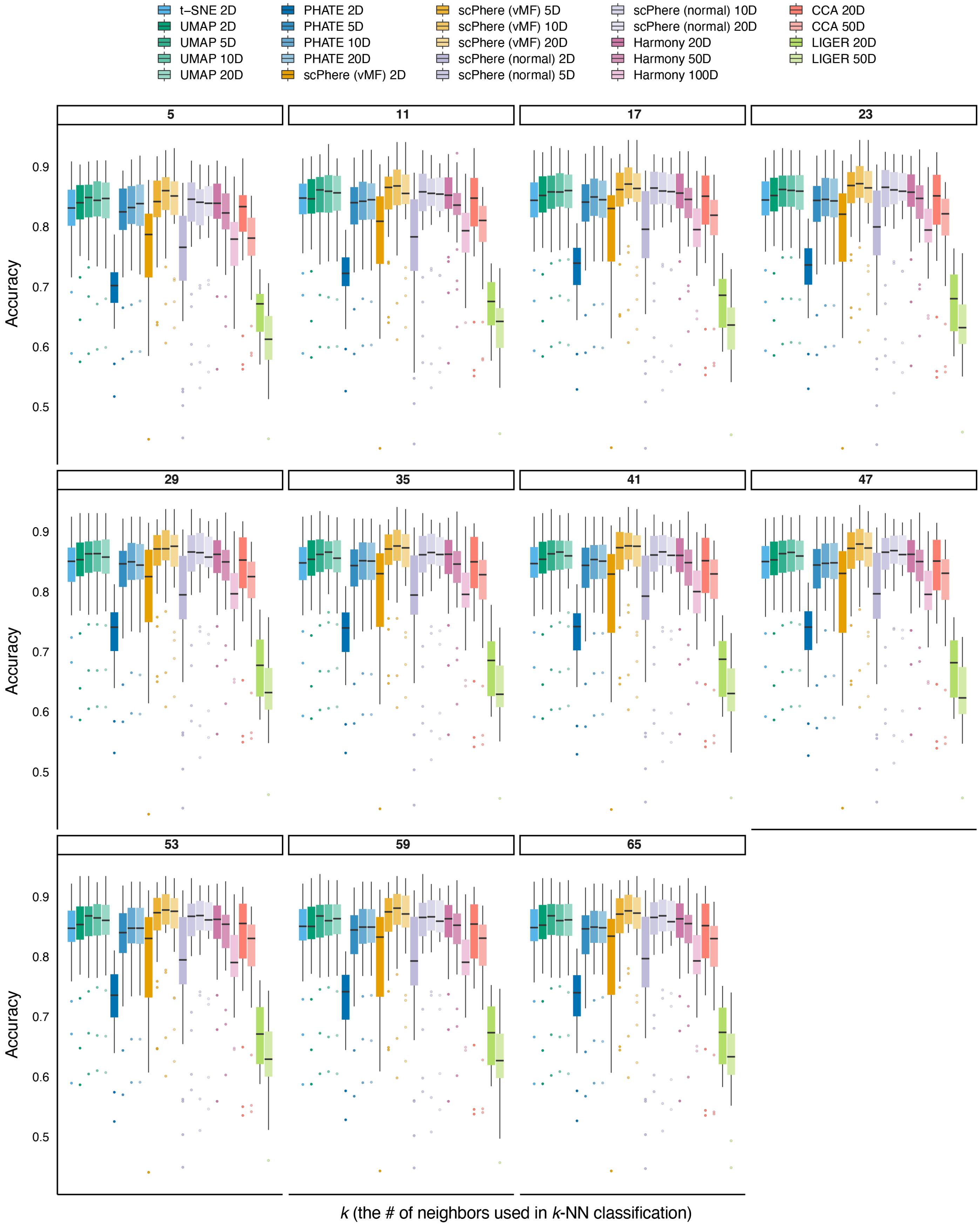
t–SNE 2D
UMAP 2D
UMAP 5D
UMAP 10D
UMAP 20D
PHATE 2D
PHATE 5D
PHATE 10D
PHATE 20D
scPhere (vMF) 2D
scPhere (vMF) 5D
scPhere (vMF) 10D
scPhere (vMF) 20D
scPhere (normal) 2D
scPhere (normal) 5D
scPhere (normal) 10D
scPhere (normal) 20D
Harmony 20D
Harmony 50D
Harmony 100D
CCA 20D
CCA 50D
LIGER 20D
LIGER 50D
5
11
17
23
29
35
41
47
53
59
65
Accuracy
0.5
0.6
0.7
0.8
0.9
k (the # of neighbors used in k-NN classification)

Supplementary Figure 6. ScPhere with hyperspherical latent spaces did favorably in batch correlation and dimensionality reduction on IBD stromal cells. k-nearest neighbor classification accuracies of stromal cell types (y-axis) for different k's (x axis), tested on the cells from one patient, after training on the cells from all other patients (n = 30 patients), taking only patient as the batch vector, using scPhere (with both hyperspherical and Euclidean latent spaces), Harmony, Seurat3 CCA, and LIGER, with different number of dimensions as inputs, as well as t-SNE, UMAP, and PHATE (each with Harmony batch-corrected 20 PCs as inputs). Boxplots denote the medians and the interquartile ranges (IQRs). The whiskers of a boxplot are the lowest datum still within 1.5 IQR of the lower quartile and the highest datum still within 1.5 IQR of the upper quartile.

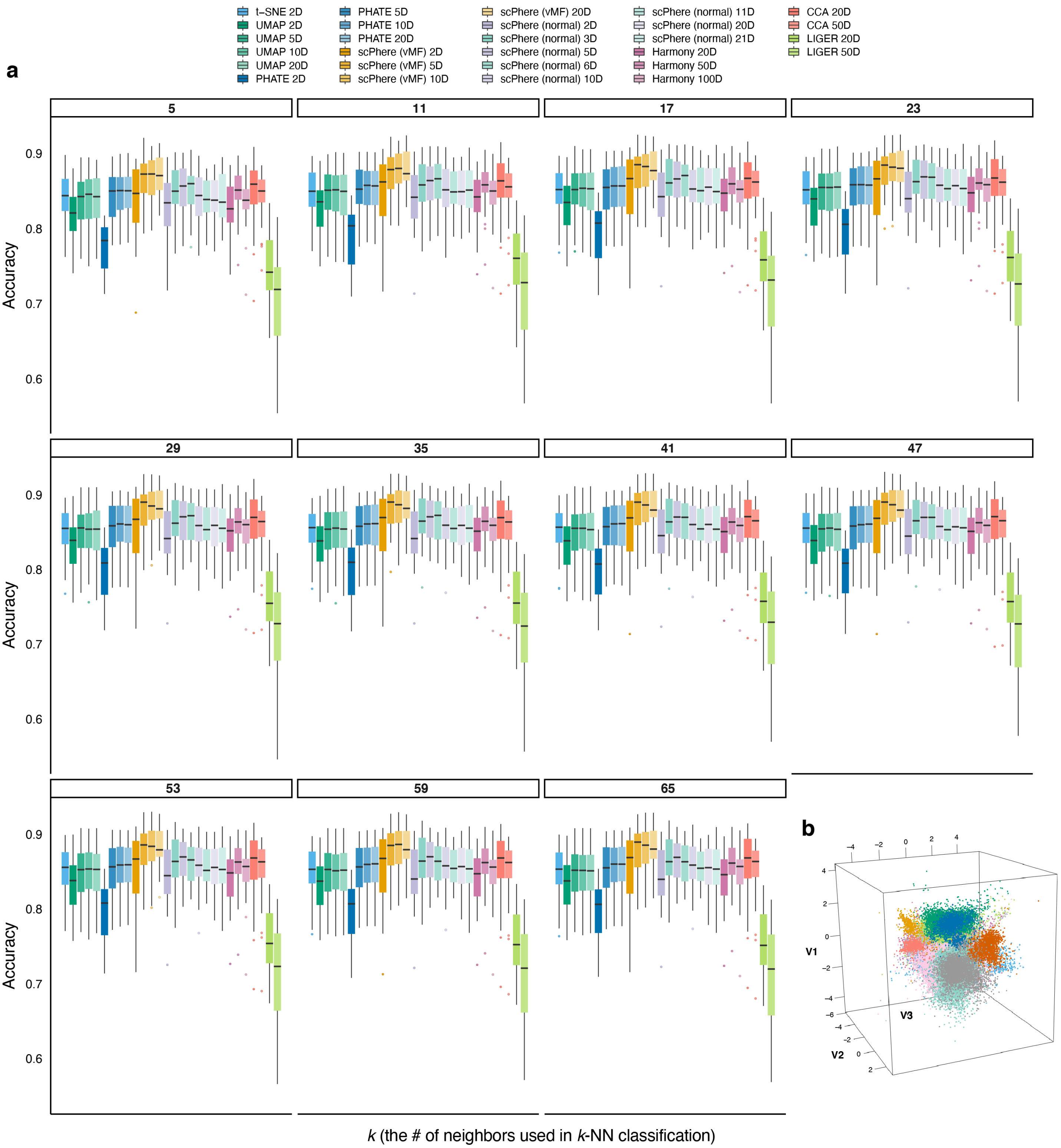

a
b
t−SNE 2D
UMAP 2D
UMAP 5D
UMAP 10D
UMAP 20D
PHATE 2D
PHATE 5D
PHATE 10D
PHATE 20D
scPhere (vMF) 2D
scPhere (vMF) 5D
scPhere (vMF) 10D
scPhere (vMF) 20D
scPhere (normal) 2D
scPhere (normal) 3D
scPhere (normal) 5D
scPhere (normal) 6D
scPhere (normal) 10D
scPhere (normal) 11D
scPhere (normal) 20D
scPhere (normal) 21D
Harmony 20D
Harmony 50D
Harmony 100D
CCA 20D
CCA 50D
LIGER 20D
LIGER 50D
5
11
17
23
29
35
41
47
53
59
65
Accuracy
k (the # of neighbors used in k-NN classification)
V1
V2
V3

Supplementary Figure 7. ScPhere with hyperspherical latent spaces did favorably in batch correlation and dimension reduction on IBD epithelial cells. (a) *k*-nearest neighbor classification accuracies of epithelial cell types (*y*-axis) for different *k*'s (*x*-axis), tested on the cells from one patient, after training on the cells from all other patients ($n = 30$ patients), using scPhere (with both hyperspherical and Euclidean latent spaces), Harmony, Seurat3 CCA, and LIGER, with different number of dimensions as inputs, as well as *t*-SNE, UMAP, and PHATE (each with Harmony batch-corrected 50 PCs as inputs). Both Seurat3 CCA and LIGER took only patient as the batch vector, while scPhere and Harmony took patient, disease, and location as the batch vector. Boxplots denote the medians and the interquartile ranges (IQRs). The whiskers of a boxplot are the lowest datum still within 1.5 IQR of the lower quartile and the highest datum still within 1.5 IQR of the upper quartile. (b) 3D scPhere latent representation with Euclidean latent spaces of the epithelial cells, coded by subset.

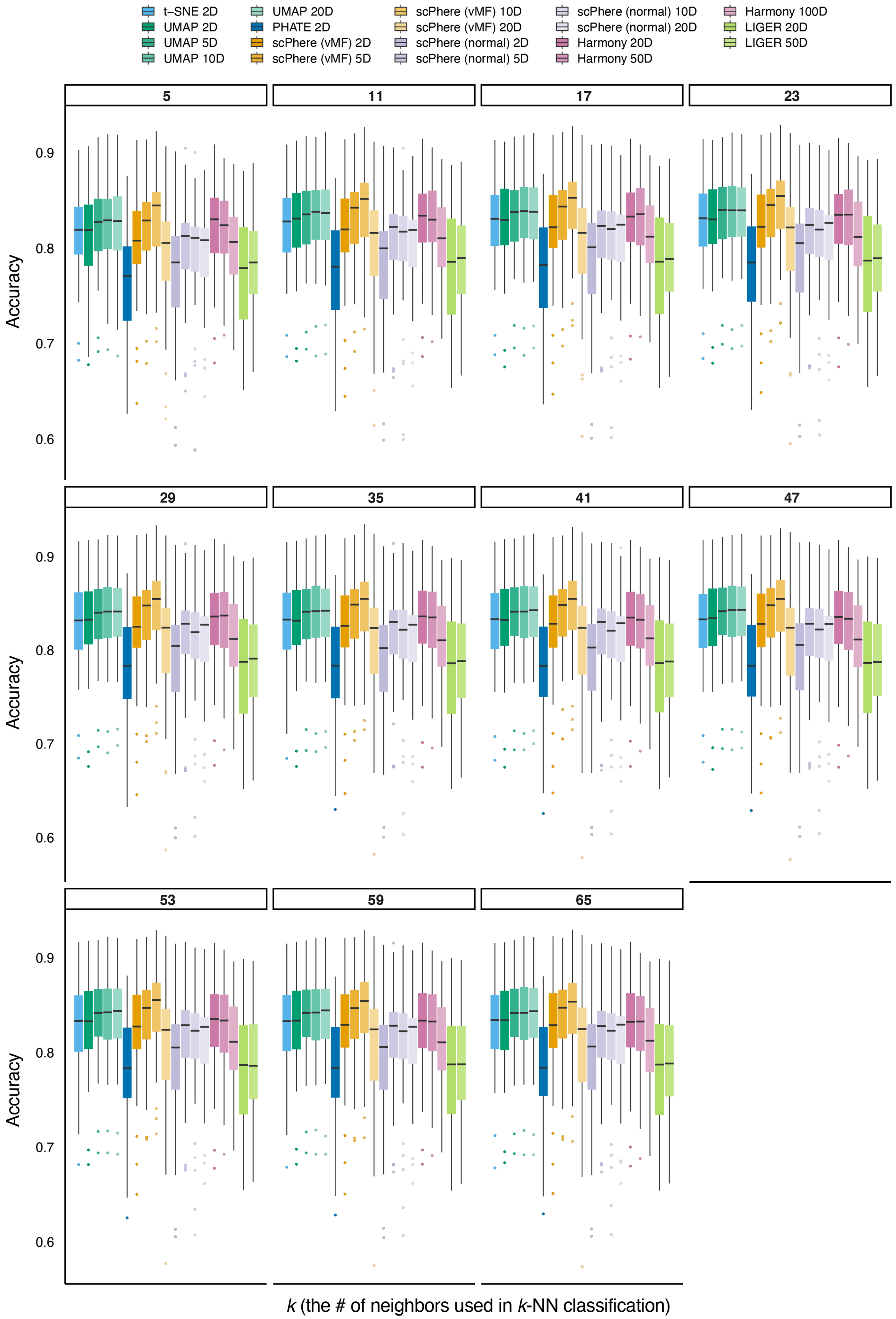
t−SNE 2D
UMAP 2D
UMAP 5D
UMAP 10D
UMAP 20D
PHATE 2D
scPhere (vMF) 2D
scPhere (vMF) 5D
scPhere (vMF) 10D
scPhere (vMF) 20D
scPhere (normal) 2D
scPhere (normal) 5D
scPhere (normal) 10D
scPhere (normal) 20D
Harmony 20D
Harmony 50D
Harmony 100D
LIGER 20D
LIGER 50D
5
11
17
23
29
35
41
47
53
59
65
Accuracy
0.6
0.7
0.8
0.9
k (the # of neighbors used in k-NN classification)

Supplementary Figure 8. ScPhere with hyperspherical latent spaces did favorably in batch correlation and dimension reduction on IBD immune cells. k-nearest neighbor classification accuracies of immune cell types (y-axis) for different k's (x-axis), tested on the cells from one patient, after training on the cells from all other patients (n = 30 patients), using scPhere (with both hyperspherical and Euclidean latent spaces), Harmony, Seurat3 CCA, and LIGER, with different number of dimensions as inputs, as well as t-SNE, UMAP, and PHATE (each with Harmony batch-corrected 50 PCs as inputs). Both Seurat3 CCA and LIGER took only patient as the batch vector, while scPhere and Harmony took patient, disease, and location as the batch vector. PHATE was only run for 2D, because of its time performance on large datasets. Boxplots denote the medians and the interquartile ranges (IQRs). The whiskers of a boxplot are the lowest datum still within 1.5 IQR of the lower quartile and the highest datum still within 1.5 IQR of the upper quartile.

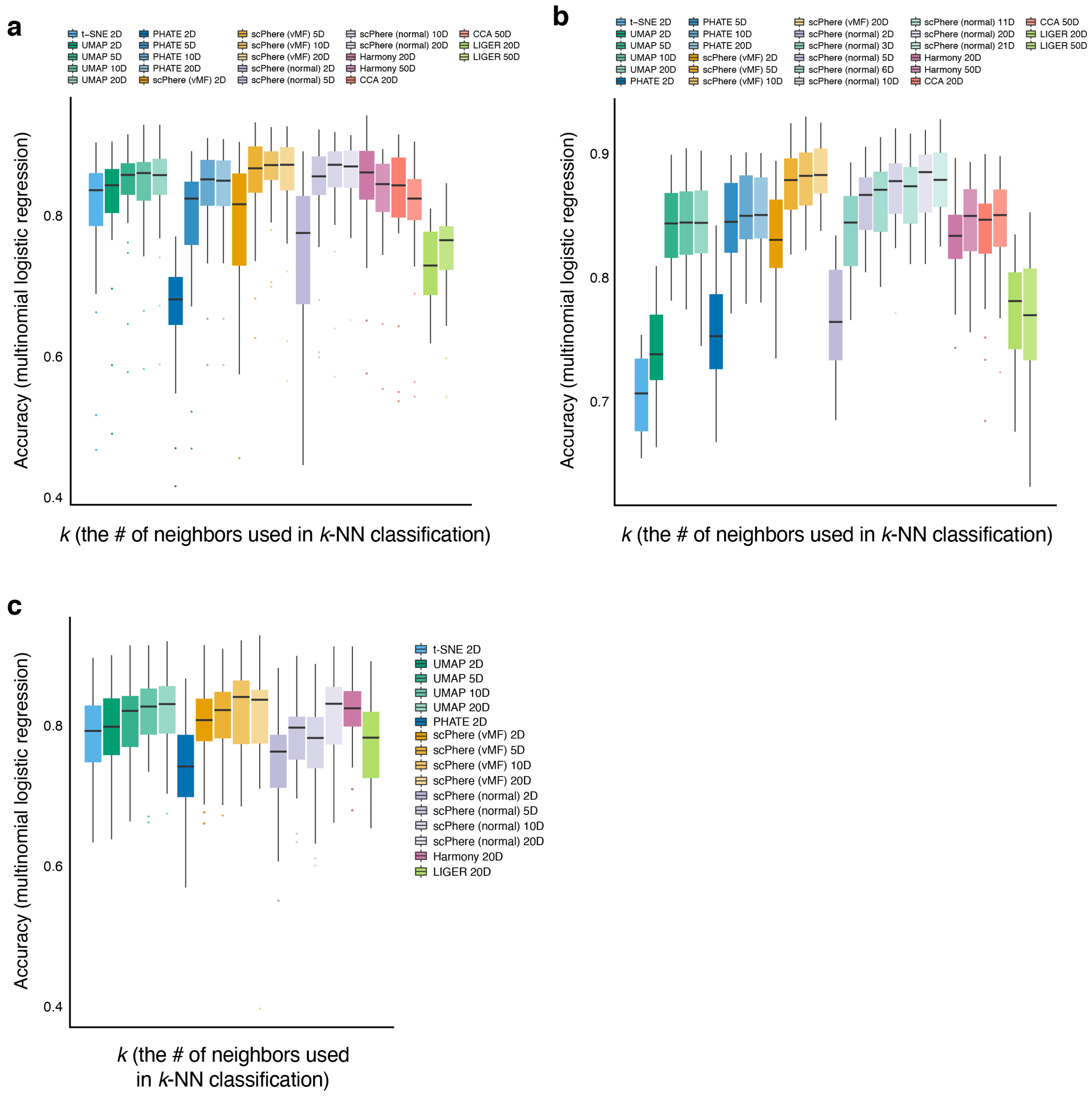

Supplementary Figure 9. ScPhere did favorably in batch correlation and dimension reduction on different IBD cell subsets based on multinomial logistic regression. Multinomial logistic regression classification accuracy (*y*-axis) of stromal (a), epithelial (b), and immune (c) cell types for different methods (*x*-axis), tested on the cells from one patient, after training on the cells from all other patients (n = 30 patients). For Seurat3 CCA and LIGER, we took the patient as the batch vector. For scPhere and Harmony, we took patient, disease, and location as the batch vector for both epithelial and immune cells, but only patient as the batch vector for the stromal cells. For immune cells, we did not calculate the logistic regression results for both Harmony 50D and LIGER 50D because there were too many parameters to fit for these cases, omitted Seurat3 CCA results because it encountered scalability issues, and ran PHATE only in the 2D case due to time performance. Boxplots denote the medians and the interquartile ranges (IQRs). The whiskers of a boxplot are the lowest datum still within 1.5 IQR of the lower quartile and the highest datum still within 1.5 IQR of the upper quartile.

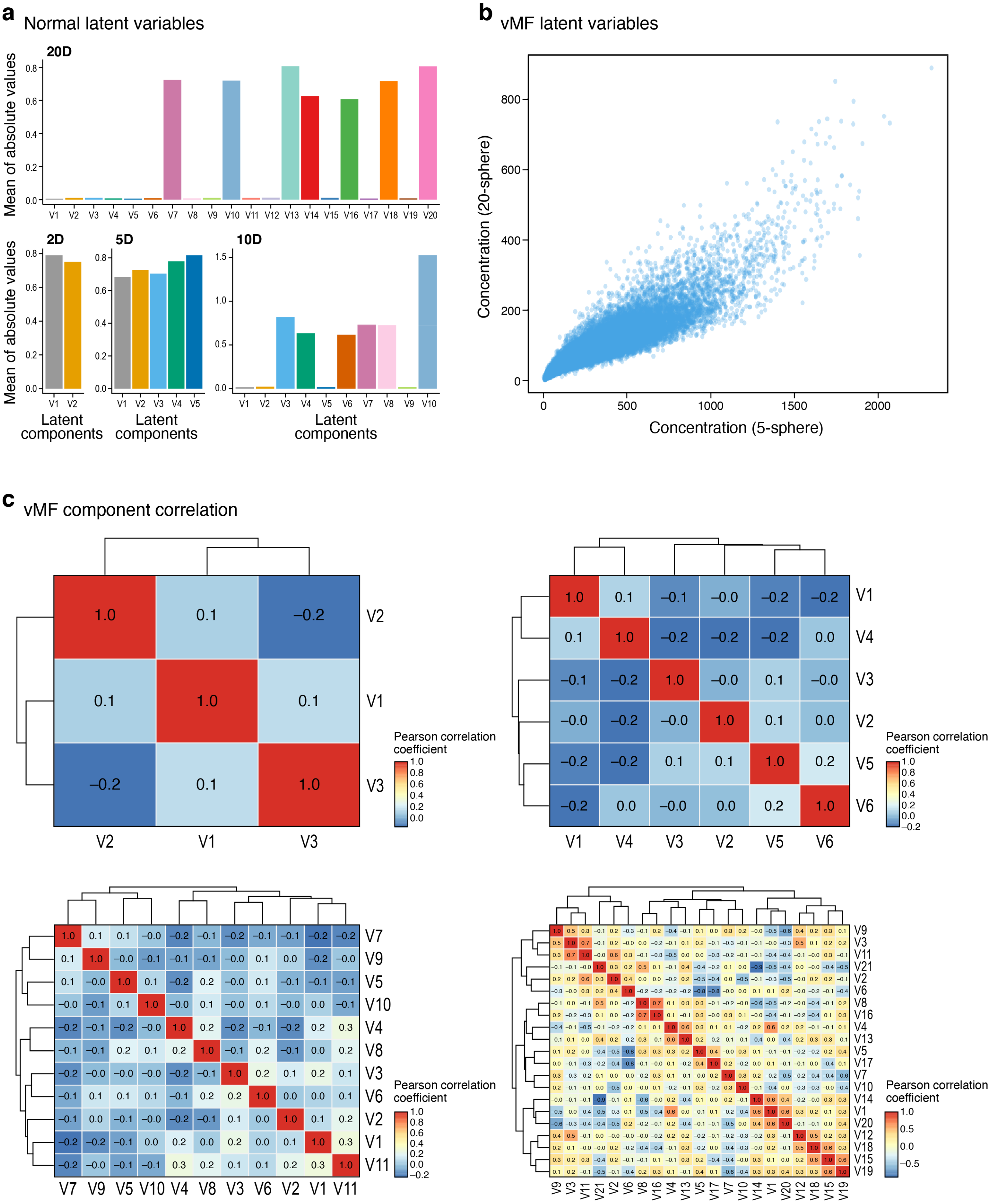

a
Normal latent variables
20D
Mean of absolute values
V1 V2 V3 V4 V5 V6 V7 V8 V9 V10 V11 V12 V13 V14 V15 V16 V17 V18 V19 V20
2D
5D
10D
Latent components
b
vMF latent variables
Concentration (20-sphere)
Concentration (5-sphere)
c
vMF component correlation
Pearson correlation coefficient

Supplementary Figure 10. The component-collapse problem using high-dimensional latent spaces. (a) Component collapse when learning Euclidean latent space with high dimensions. The means of the absolute values (*y*-axis) of each latent component (*x*-axis) learned by scPhere with Euclidean latent space with different numbers of dimensions across all the IBD epithelial cells. (b) vMF concentration parameters of each cell (dot) when using 5D (*x*-axis) or 20D (*y*-axis) hyperspherical latent spaces. High concentrations reflect low uncertainties. (c) Increased correlation between components when learning hyperspherical latent space with higher dimensions. Pearson correlation coefficients (color bars) between any two components of the learned hyperspherical latent variables across cells for models with 2, 5, 10, or 20-spheres.

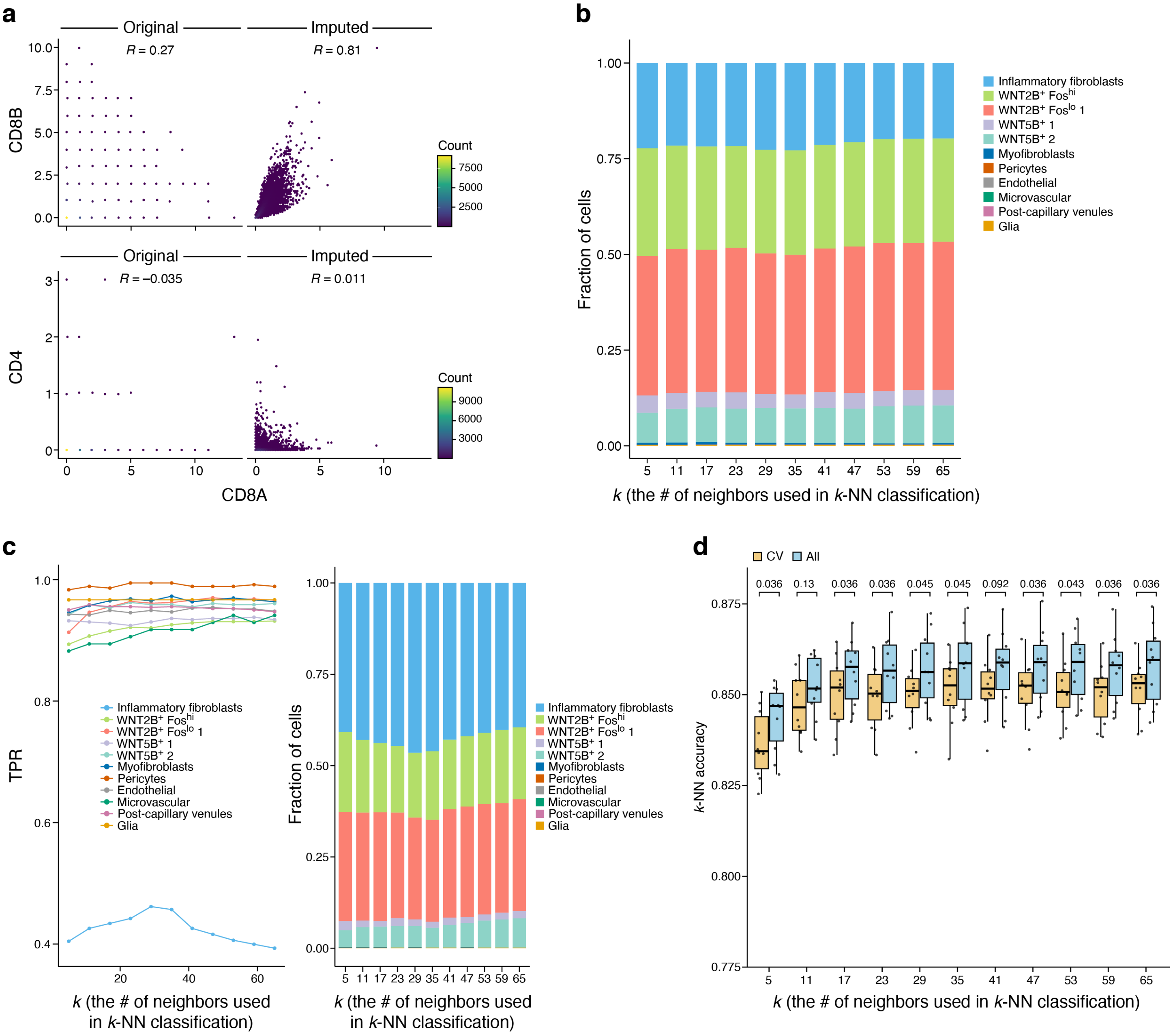
a
Original
Imputed
R = 0.27
R = 0.81
R = −0.035
R = 0.011
CD8B
CD4
CD8A
Count
b
Fraction of cells
k (the # of neighbors used in k-NN classification)
Inflammatory fibroblasts
WNT2B+ Foshi
WNT2B+ Foslo 1
WNT5B+ 1
WNT5B+ 2
Myofibroblasts
Pericytes
Endothelial
Microvascular
Post-capillary venules
Glia
c
TPR
Fraction of cells
k (the # of neighbors used in k-NN classification)
d
CV
All
k-NN accuracy
0.036
0.13
0.036
0.036
0.045
0.045
0.092
0.036
0.043
0.036
0.036
k (the # of neighbors used in k-NN classification)

Supplementary Figure 11. ScPhere can denoise data, pinpoint the impacted cell types by disease, and classify cells from unseen test data. (a) Imputation by scPhere model. Comparison of *CD8A* (*x*-axis) vs. *CD8B* (*y*-axis, top) or CD4 (*y*-axis, bottom) expression in $CD8^+$ T cells from the UC immune cells, based on original counts (left) or scPhere decoder outputs (right). Pearson correlation coefficients labeled on top. (b) Classification-based identification of cells impacted by biological factors. Fraction of inflammatory fibroblasts (*y*-axis) assigned to different cell types (colored bars) by a *k*-nearest neighbor classification of UC stromal cells from inflamed biopsies trained using cells from healthy and non-inflamed tissues after using both patient and disease (healthy, non-inflamed, inflamed) as the batch vector. (c) As in (b) and Fig. 4c, but only using high-confident cells that were corrected classified using scPhere embedding when taking patient as the only batch vector. (d) *k*-NN classification accuracy (*y* axis) of UC stromal cells in 10-fold cross-validation with 5D hyperspheres for different values of *k* (*x* axis) with classifiers trained on either data mapped on a 5D hypersphere (orange; variable gene selection, scPhere modeling fitting, and *k*-NN classifier training were done in each fold) or when the 5D representation of cells was pre-computed by learning a scPhere model using all the cells (blue). Adjusted *p*-values (FDR, paired Wilcoxon rank sum test, two-sided) comparing the classification accuracies are at the top. Boxplots denote the medians and the interquartile ranges (IQRs). The whiskers of a boxplot are the lowest datum still within 1.5 IQR of the lower quartile and the highest datum still within 1.5 IQR of the upper quartile.

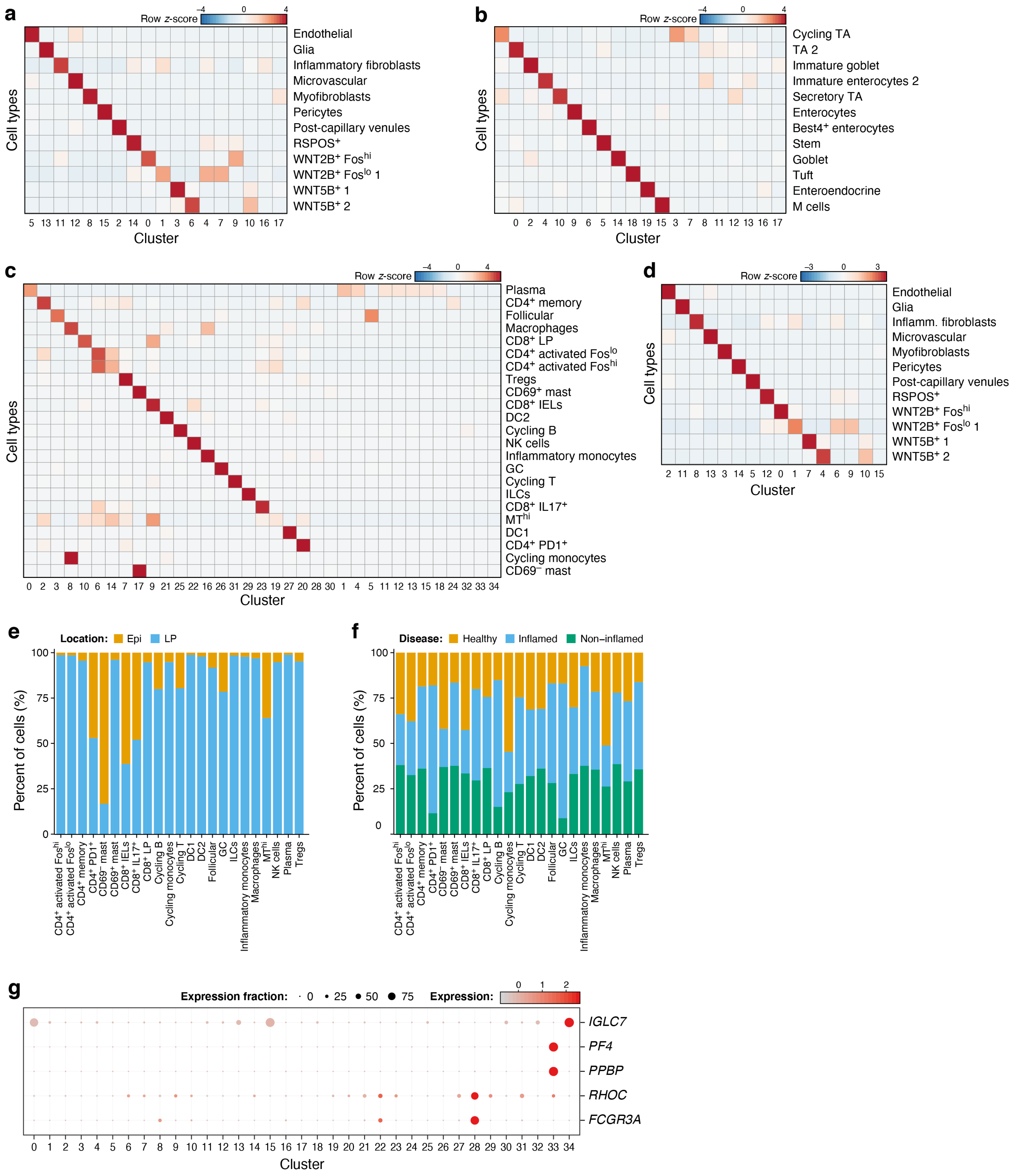
a
Row z-score
−4
0
4
Endothelial
Glia
Inflammatory fibroblasts
Microvascular
Myofibroblasts
Pericytes
Post-capillary venules
RSPOS+
WNT2B+ Foshi
WNT2B+ Foslo 1
WNT5B+ 1
WNT5B+ 2
Cell types
5 13 11 12 8 15 2 14 0 1 3 6 4 7 9 10 16 17
Cluster
b
Row z-score
−4
0
4
Cycling TA
TA 2
Immature goblet
Immature enterocytes 2
Secretory TA
Enterocytes
Best4+ enterocytes
Stem
Goblet
Tuft
Enteroendocrine
M cells
Cell types
0 2 4 10 9 6 5 14 18 19 15 3 7 8 11 12 13 16 17
Cluster
c
Row z-score
−4
0
4
Plasma
CD4+ memory
Follicular
Macrophages
CD8+ LP
CD4+ activated Foslo
CD4+ activated Foshi
Tregs
CD69+ mast
CD8+ IELs
DC2
Cycling B
NK cells
Inflammatory monocytes
GC
Cycling T
ILCs
CD8+ IL17+
MThi
DC1
CD4+ PD1+
Cycling monocytes
CD69− mast
Cell types
0 2 3 8 10 6 14 7 17 9 21 25 22 16 26 31 29 23 19 27 20 28 30 1 4 5 11 12 13 15 18 24 32 33 34
Cluster
d
Row z-score
−3
0
3
Endothelial
Glia
Inflamm. fibroblasts
Microvascular
Myofibroblasts
Pericytes
Post-capillary venules
RSPOS+
WNT2B+ Foshi
WNT2B+ Foslo 1
WNT5B+ 1
WNT5B+ 2
Cell types
2 11 8 13 3 14 5 12 0 1 7 4 6 9 10 15
Cluster
e
Location:
Epi
LP
Percent of cells (%)
100
75
50
25
0
CD4+ activated Foshi
CD4+ activated Foslo
CD4+ memory
CD4+ PD1+
CD69− mast
CD69+ mast
CD8+ IELs
CD8+ IL17+
CD8+ LP
Cycling B
Cycling monocytes
Cycling T
DC1
DC2
Follicular
GC
ILCs
Inflammatory monocytes
Macrophages
MThi
NK cells
Plasma
Tregs
f
Disease:
Healthy
Inflamed
Non−inflamed
Percent of cells (%)
100
75
50
25
0
CD4+ activated Foshi
CD4+ activated Foslo
CD4+ memory
CD4+ PD1+
CD69− mast
CD69+ mast
CD8+ IELs
CD8+ IL17+
CD8+ LP
Cycling B
Cycling monocytes
Cycling T
DC1
DC2
Follicular
GC
ILCs
Inflammatory monocytes
Macrophages
MThi
NK cells
Plasma
Tregs
g
Expression fraction:
0
25
50
75
Expression:
0
1
2
IGLC7
PF4
PPBP
RHOC
FCGR3A
0 1 2 3 4 5 6 7 8 9 10 11 12 13 14 15 16 17 18 19 20 21 22 23 24 25 26 27 28 29 30 31 32 33 34
Cluster

Supplementary Figure 12. Clustering colon mucosal cells in the latent representations of 5D hyperspheres. (a–d) Effecting clustering on hypersphere. Overlap (number of cells, row-centered and scaled *Z*-score, color bar) of cluster membership between the clusters in the original study (rows) and those obtained by Louvain clustering on the cell embedding to a hypersphere (columns) for stromal (a), epithelial (b), or immune (c) cells with 5D hypersphere, or stromal cells (d) with a 10D hypersphere. (e, f) Location and disease distribution vary across immune cell clusters. Percent of cells (*y*-axis) from a given location (e, Epi or LP) or disease state (f, healthy, uninflamed, or inflamed) in each immune cell subset annotated in the original study (*x*-axis). (g) New immune clusters have distinctive expression markers. Fraction of expressing cells (dot size) and mean level of expression in expressing cells (dot color, row *Z*-score of log(transcripts per 10,000)) for selected marker genes (rows) differentially expressed in small clusters of immune cells (cluster 28, 33, and 34, columns).

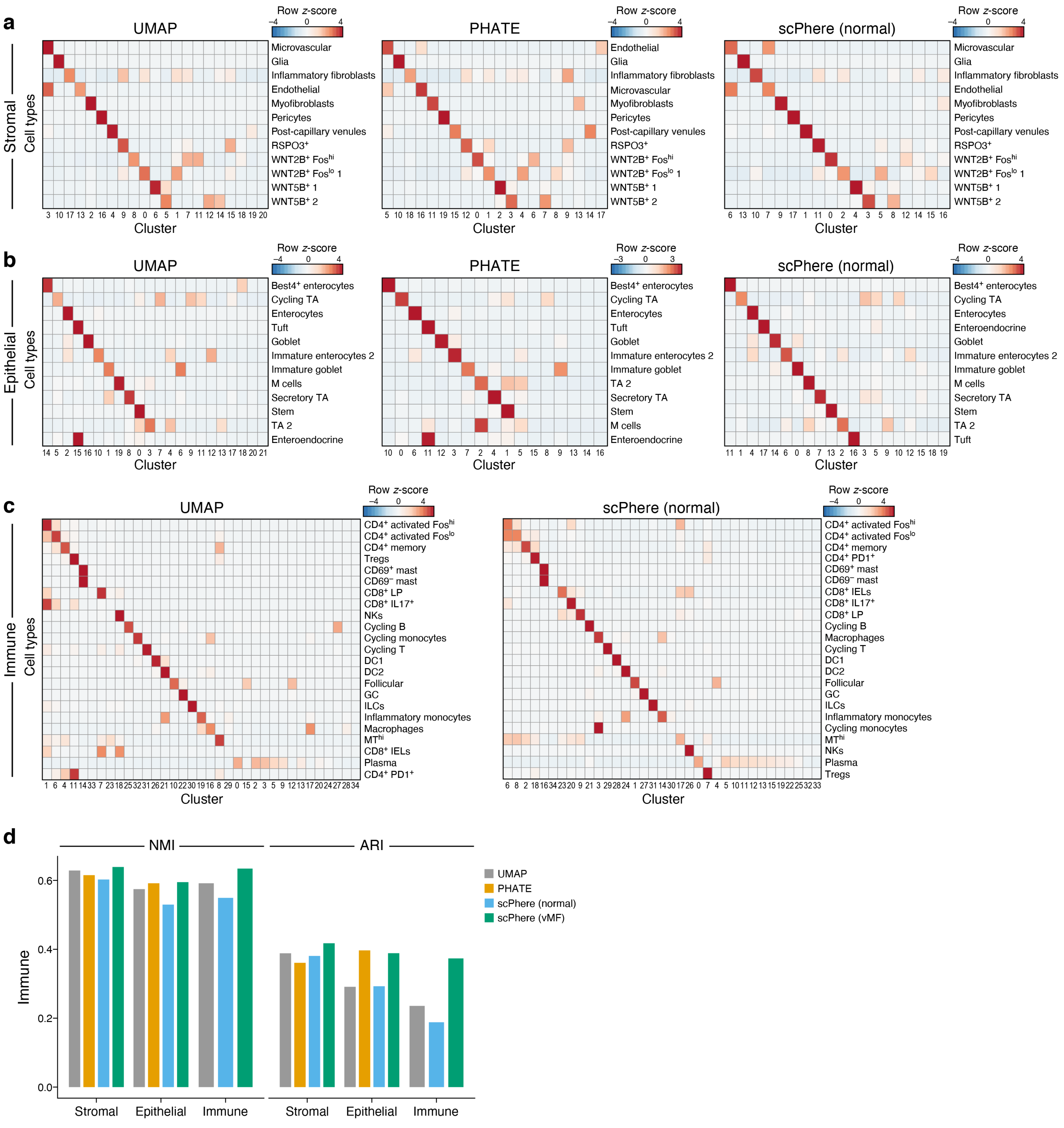

Supplementary Figure 13. Comparison of clustering on embeddings from UMAP, PHATE, and scPhere with Euclidean latent spaces. (a–c) Clustering using UMAP, PHATE, and scPhere embeddings (with Euclidean latent spaces). Overlap (number of cells, row-centered and scaled *Z*-score, color bar) of cluster membership between the clusters in the original study (rows) and those obtained by Louvain clustering of 5D representations of stromal (a), epithelial (b), and immune (c) cells from UMAP, PHATE, and scPhere. (d) Cluster Normalized Mutual Information (NMI) and Adjusted Rand Index (ARI) metrics, for the clustering in a–c. For immune cells, PHATE results are not included due to time performance.

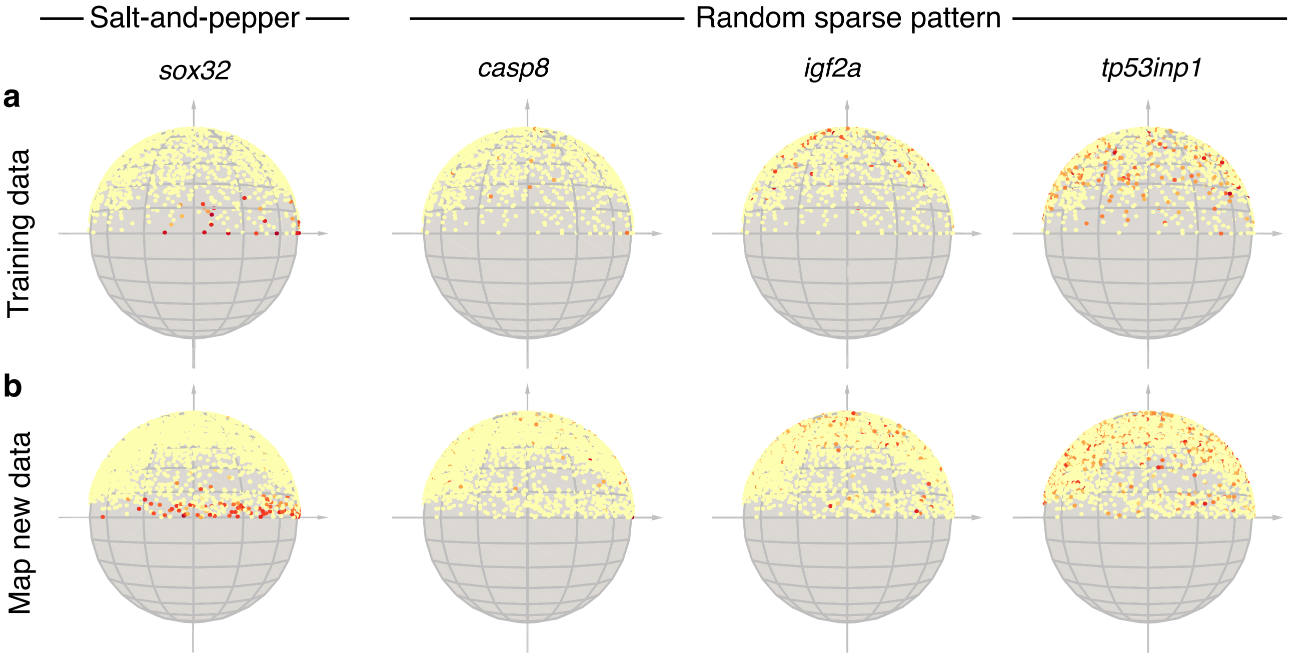

Supplementary Figure 14. ScPhere infers ‘salt-and-pepper’ and random sparse spatial patterns correctly. ScPhere inferred spatial locations of 1,406 cells from the training data (a) or 3,820 cells from another three testing batches (b) colored by expression (log (transcripts per 10,000), color bar) of marked genes representative of cells distributed in ‘salt-and-pepper’ patterns or in ‘random sparse’ patterns (characteristic of ‘apoptotic-like’ cells). (The genes were not among the 11 landmarks used in training.)

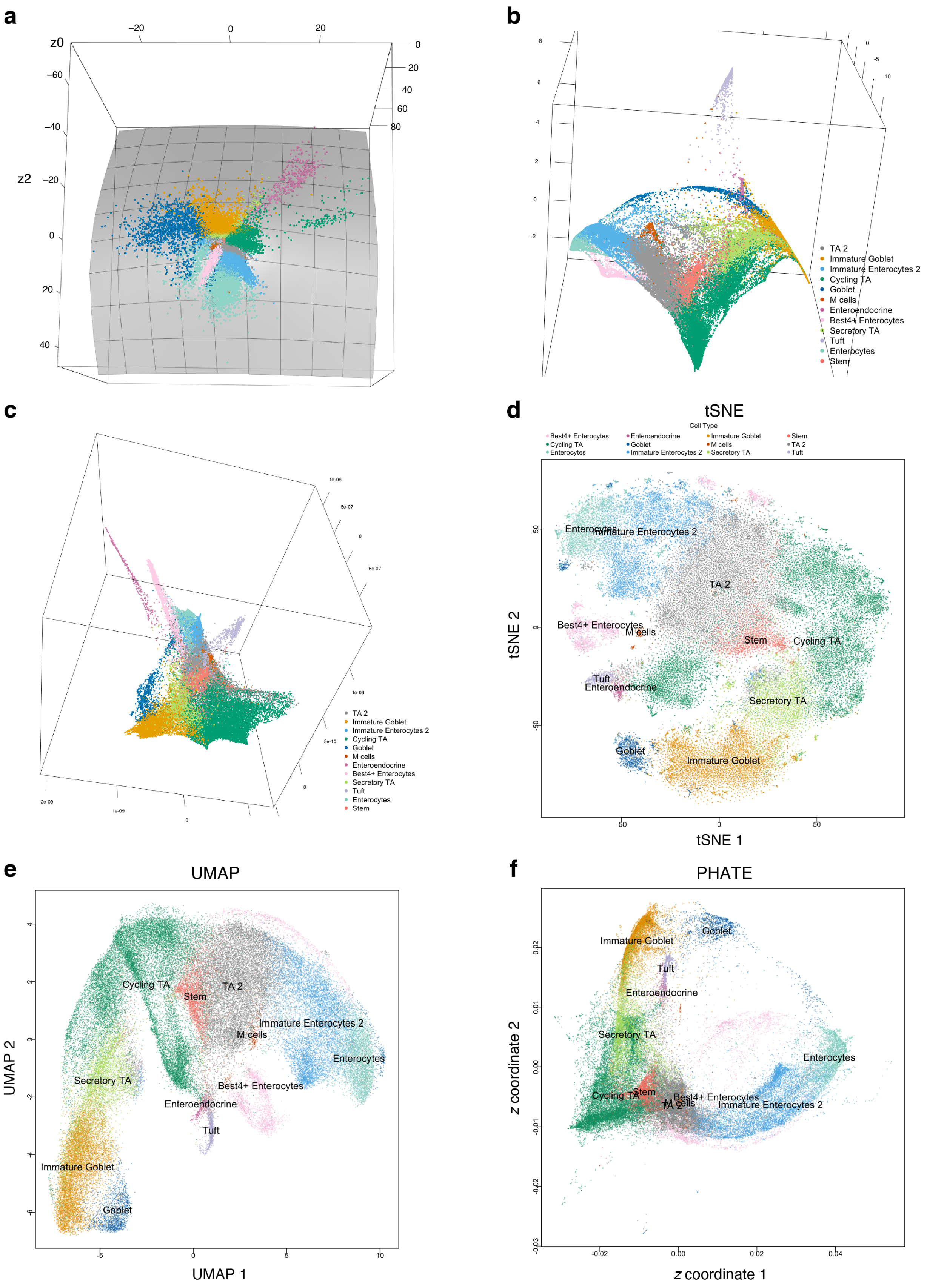

Supplementary Fig. 15. Visualizing epithelial cell differentiation by different embeddings. Embeddings of epithelial cells colored by cell type in either the 2D hyperbolic space of the Lorentz model (a), PHATE multidimensional scaling with the 5D representations of cells in either the hyperbolic space of the Lorentz model (b) or the Euclidean space (c) as inputs, *t*-SNE (d), UMAP (e), or PHATE (f).

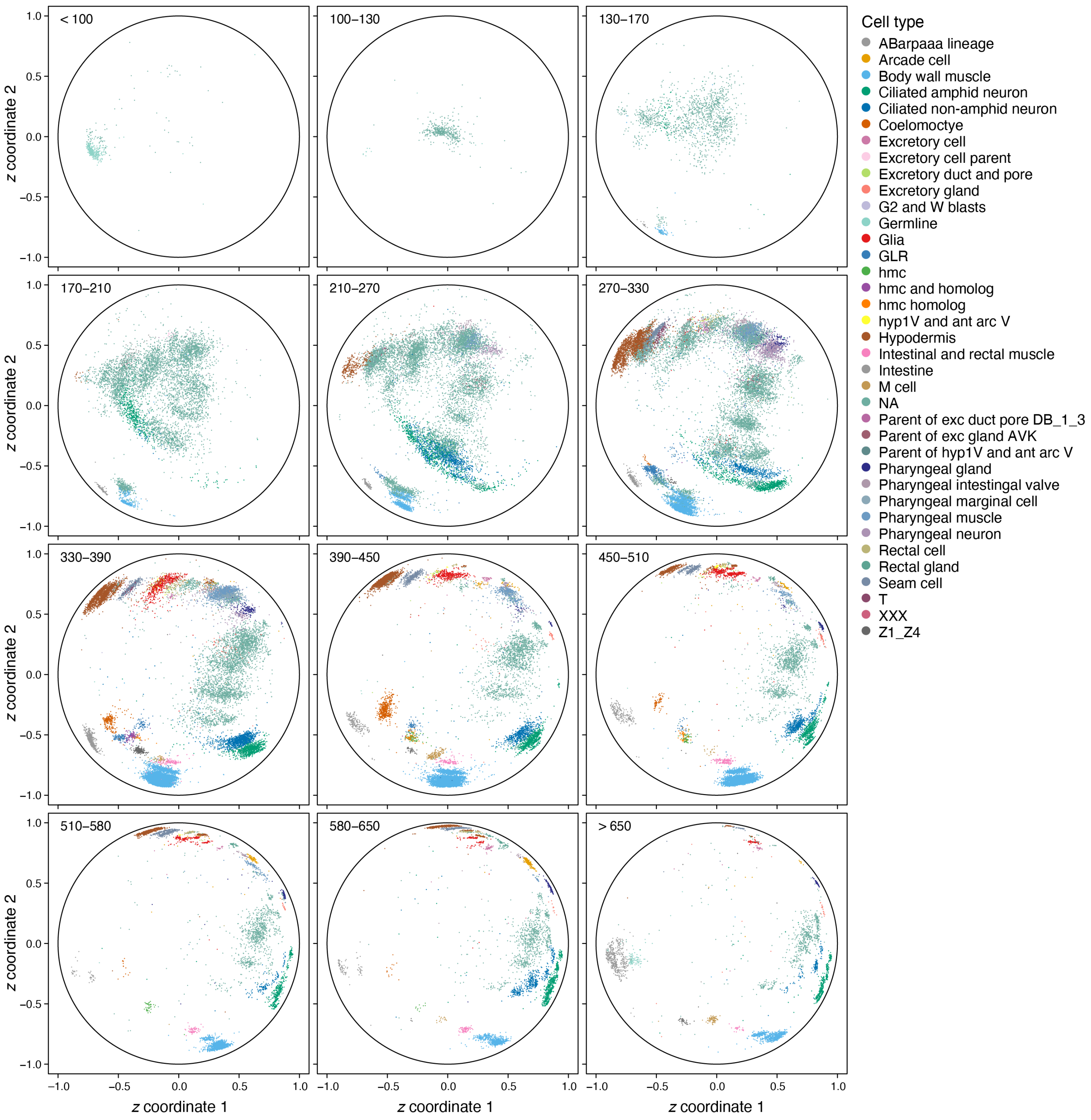

Supplementary Figure 16. Poincaré disk embedding highlights the progression of *C. elegans* embryonic cells in time. Embedding of all *C. elegans* embryonic cells in a Poincaré disk (as in Fig. 6c, d), with each panel showing only the cells from one of 12 embryonic time bins (labeled in top left corner). Cells are colored by annotated cell types.

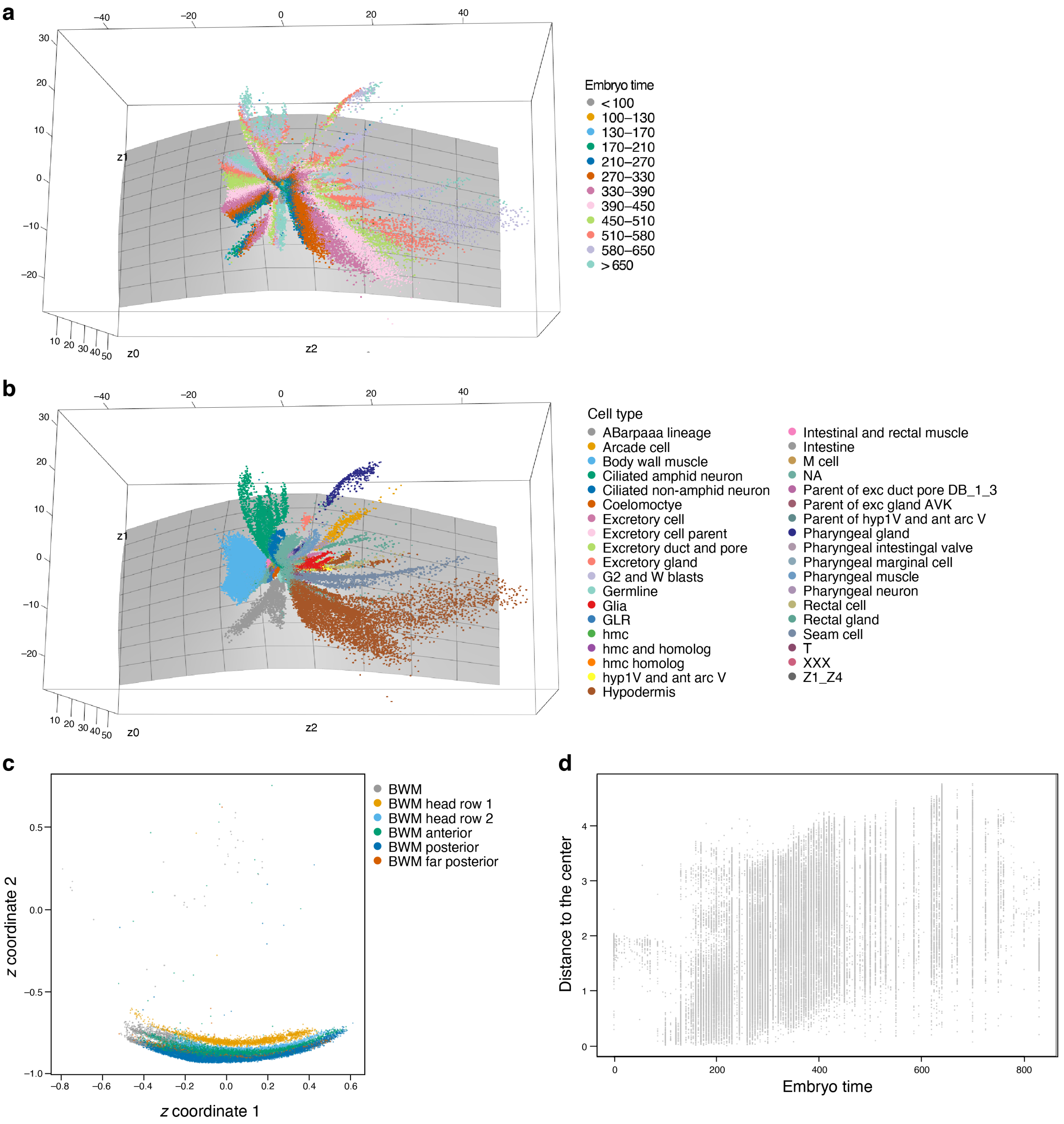

Supplementary Figure 17. Studying cell differentiation by embedding *C. elegans* embryonic cells into a hyperbolic space. (a, b) Embedding of *C. elegans* embryonic cells in the Lorentz model, colored by (a) embryo time or (b) cell type. (c) Fine resolution of cell differentiation and location. Embedding of *C. elegans* embryonic cells in Poincaré disk coordinates (as in Fig. 6c, d), but showing only body wall muscle (BWM) cells, colored by lineage/location from first row and second row BWMs (MS lineage) to posterior BWMs (C lineage). (d) Distance from Poincaré disk center as pseudotime. Relating the embryo real time (y-axis) for each cell (dot) and the cell's distance to the center of the Poincaré disk (x-axis) (cells from time point 100–130 were positioned in the center, at distance 0).

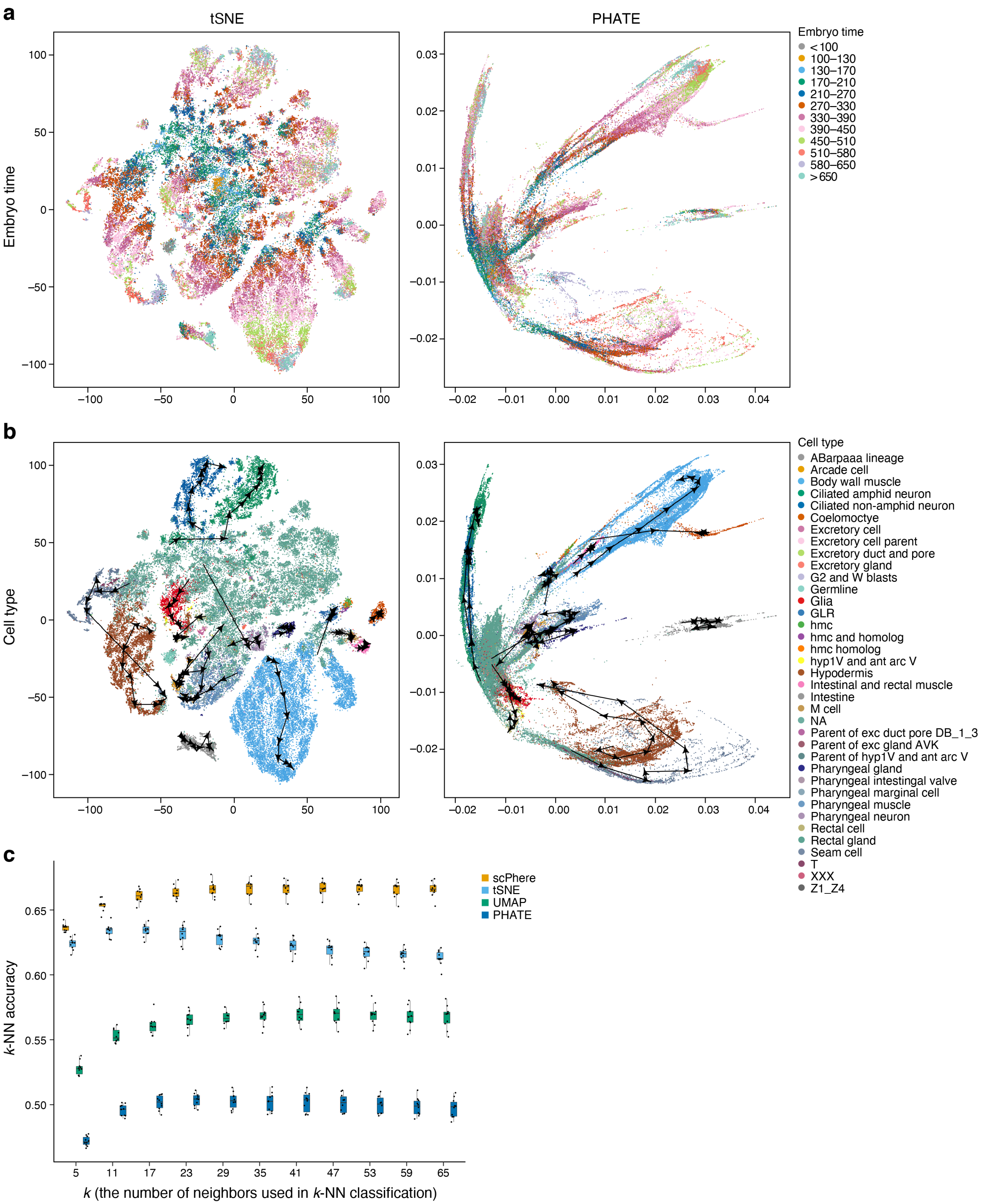
a
tSNE
PHATE
Embryo time
Embryo time
< 100
100–130
130–170
170–210
210–270
270–330
330–390
390–450
450–510
510–580
580–650
> 650
b
Cell type
Cell type
ABarpaaa lineage
Arcade cell
Body wall muscle
Ciliated amphid neuron
Ciliated non-amphid neuron
Coelomoctye
Excretory cell
Excretory cell parent
Excretory duct and pore
Excretory gland
G2 and W blasts
Germline
Glia
GLR
hmc
hmc and homolog
hmc homolog
hyp1V and ant arc V
Hypodermis
Intestinal and rectal muscle
Intestine
M cell
NA
Parent of exc duct pore DB_1_3
Parent of exc gland AVK
Parent of hyp1V and ant arc V
Pharyngeal gland
Pharyngeal intestingal valve
Pharyngeal marginal cell
Pharyngeal muscle
Pharyngeal neuron
Rectal cell
Rectal gland
Seam cell
T
XXX
Z1_Z4
c
scPhere
tSNE
UMAP
PHATE
k-NN accuracy
k (the number of neighbors used in k-NN classification)

Supplementary Figure 18. Embedding cells in hyperbolic spaces retains embryonic time continuity. (a, b) *C. elegans* single cell profiles (dots) embedded by *t*-SNE (left) or PHATE (right) (with 50 Harmony batch corrected PCs as inputs) and colored by time (a) or cell type (b). (c) *k*-NN classification accuracies of embryonic times (*y*-axis) for different *k*'s (*x*-axis) in a 10-fold cross validation analysis for each embedding method ($n = 10$). Boxplots denote the medians and the interquartile ranges (IQRs). The whiskers of a boxplot are the lowest datum still within 1.5 IQR of the lower quartile and the highest datum still within 1.5 IQR of the upper quartile.

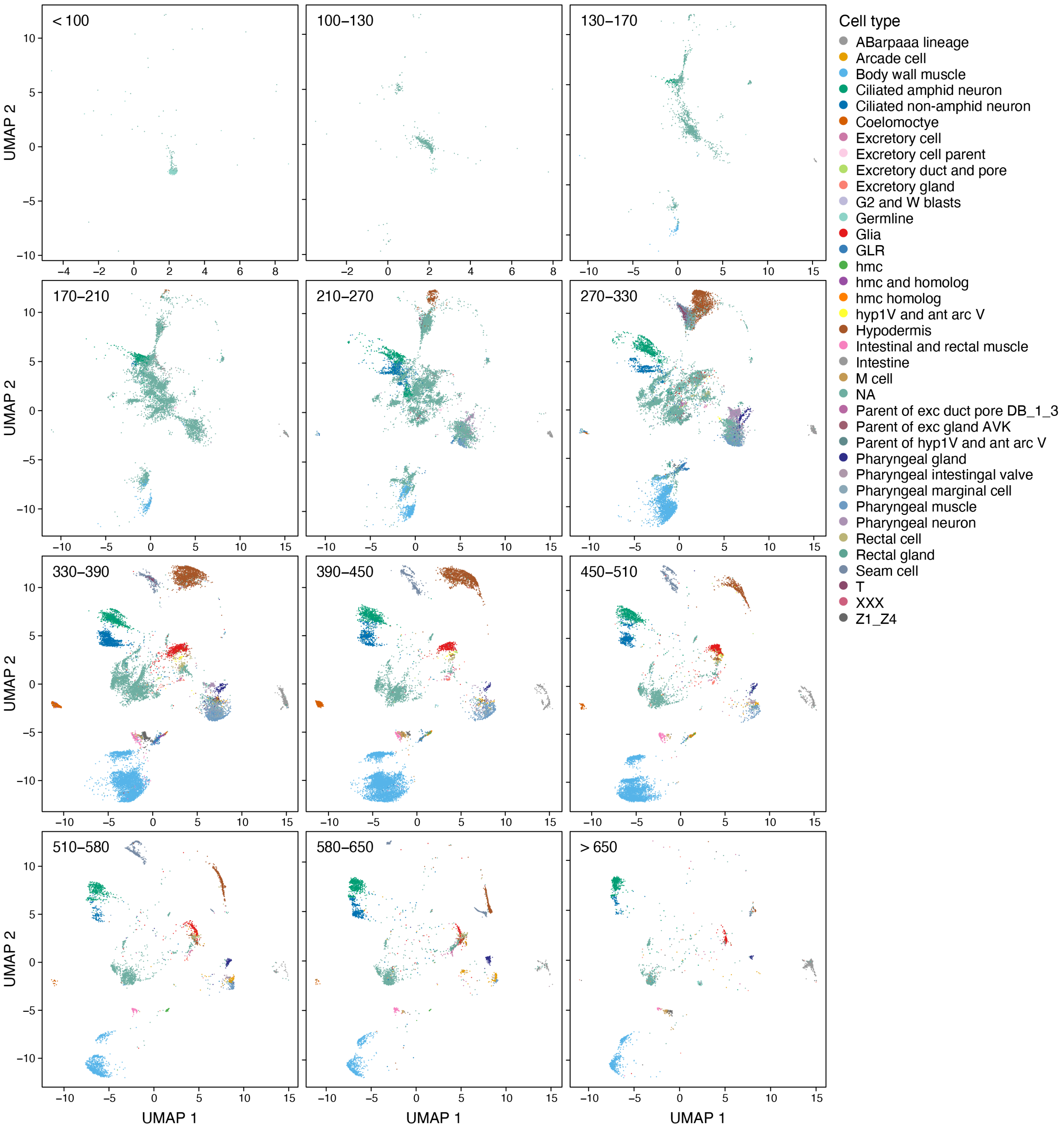

Supplementary Figure 19. UMAP embedding of *C. elegans* embryonic cells along time. Embedding of all *C. elegans* embryonic cells in a UMAP (as in Fig. 6e, f), with each panel showing only the cells from one of 12 embryonic time bins (labeled in top left), colored by annotated cell types.

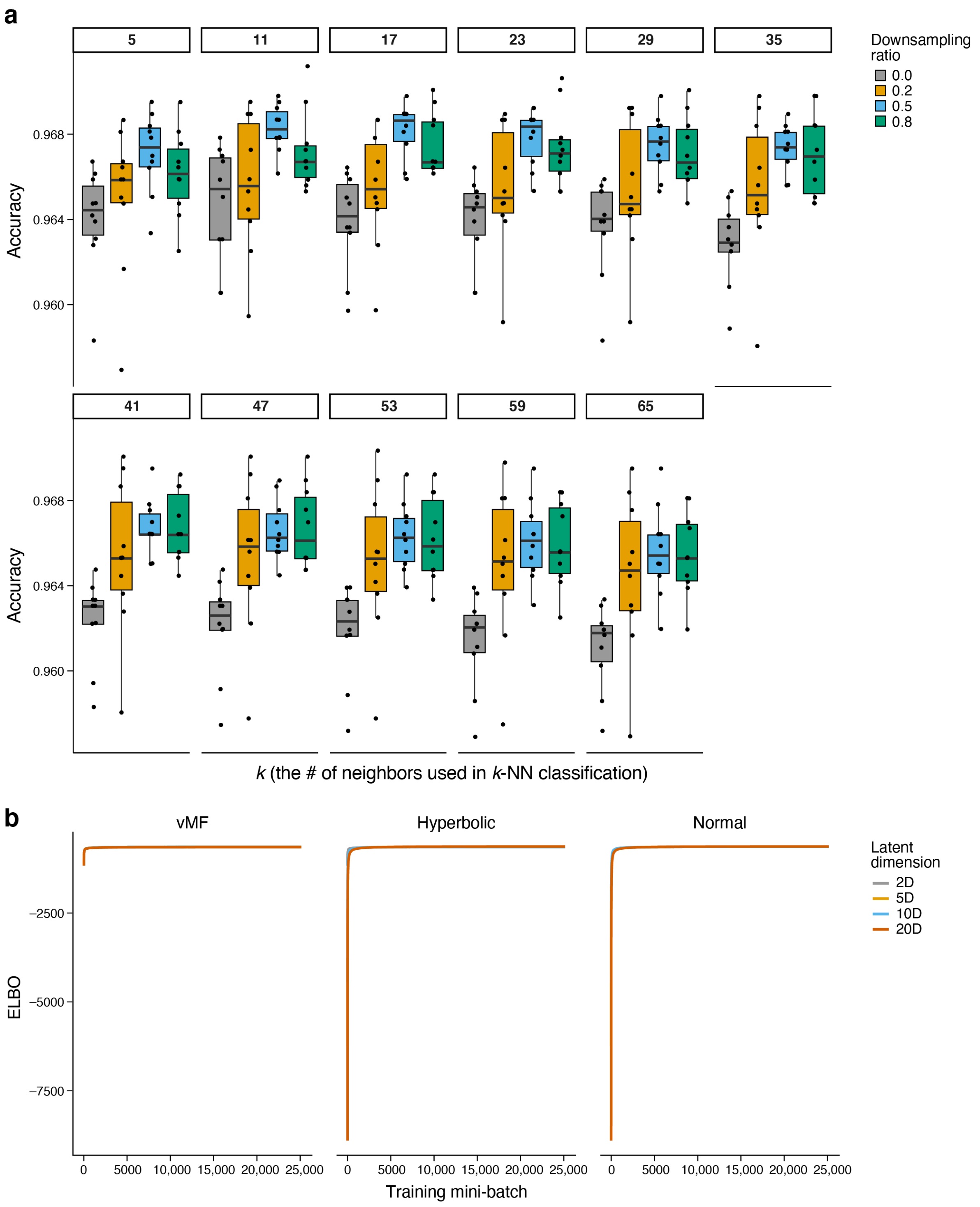

Supplementary Figure 20. ScPhere performance with a penalty term and stability in training. (a) Penalty term improves scPhere performance. *K*-NN accuracies (*y*-axis) at different down-sampling ratios and without a penalty term (i.e., down-sampling ratio = 0) in a 10-fold cross validation analysis ($n = 10$) for different *k*'s, Boxplots denote the medians and the interquartile ranges (IQRs). The whiskers of a boxplot are the lowest datum still within 1.5 IQR of the lower quartile and the highest datum still within 1.5 IQR of the upper quartile. (b) Stability and rapid convergence in training. Average ELBO changes (*y*-axis) with training mini-batches for hyperspherical, hyperbolic, and Euclidean latent spaces.
