# Supplementary material for: Deep generative model embedding of single-cell RNA-Seq profiles on hyperspheres and hyperbolic spaces
Source: Nat Commun. 2021 May 5;12:2554. doi: 10.1038/s41467-021-22851-4 (PMC8099904; doi:10.1038/s41467-021-22851-4)
Supplement: Supplementary file 3 — Description of Additional Supplementary Files [file 41467_2021_22851_MOESM3_ESM.pdf]

Description of Additional Supplementary Files

File Name: Supplementary Movie 1

Description: **Embedding of stromal cells from human colon mucosa on the surface of the unit sphere, taking patient as the batch vector.**

Cells (dot) are color-coded by type. Annotations are marked adjacent to the corresponding cells.

File Name: Supplementary Movie 2

Description: **Embedding stromal cells from human colon mucosa on the surface of the unit sphere, taking both patient and disease as the batch vector.**

Cells (dot) are color-coded by type. Annotations are marked adjacent to the corresponding cells. We did not include anatomical regions in the batch vector, because most cells were from the lamina propria fraction.

File Name: Supplementary Movie 3

Description: **Embedding epithelial cells from human colon mucosa on the surface of the unit sphere, taking patient, disease status, and anatomical region as the batch vector.**

Cells (dot) are color-coded by type. Annotations are marked adjacent to the corresponding cells.

File Name: Supplementary Movie 4

Description: **Embedding all cells from human colon mucosa on the surface of the unit sphere, taking patient, disease status, and anatomical region as the batch vector.**

Cells (dot) are color-coded by type. Annotations are marked adjacent to the corresponding cells.

File Name: Supplementary Movie 5

Description: **Clustering the 5D latent representations of immune cells from human colon mucosa.**

Embedding immune cells on the surface of a unit sphere, taking patient, disease status, and anatomical region as the batch vector. Cells (dot) are color-coded by cluster membership numbers (and assigned cell types) as determined by Louvain clustering (resolution=1.2, the number of nearest neighbors=25) of the latent representations from embedding the cells on the surface of a 5D hypersphere.

File Name: Supplementary Software 1
Description: **The scPhere python package developed in this study.**

The scPhere package, written in Python and TensorFlow, learn the low-dimensional latent structures in single-cell RNA-Seq data.
