# Supplementary material for: Deep generative model embedding of single-cell RNA-Seq profiles on hyperspheres and hyperbolic spaces
Source: Nat Commun. 2021 May 5;12:2554. doi: 10.1038/s41467-021-22851-4 (PMC8099904; doi:10.1038/s41467-021-22851-4)
Supplement: Supplementary file 10 — Reporting Summary [file 41467_2021_22851_MOESM10_ESM.pdf]

nature research

Corresponding author(s): Jiarui Ding and Aviv Regev

Last updated by author(s): Mar 15, 2021

Reporting Summary

Nature Research wishes to improve the reproducibility of the work that we publish. This form provides structure for consistency and transparency in reporting. For further information on Nature Research policies, see Authors & Referees and the Editorial Policy Checklist .

Statistics

For all statistical analyses, confirm that the following items are present in the figure legend, table legend, main text, or Methods section.

n/a	Confirmed	
☐	☒	The exact sample size (*n*) for each experimental group/condition, given as a discrete number and unit of measurement
☐	☒	A statement on whether measurements were taken from distinct samples or whether the same sample was measured repeatedly
☐	☒	The statistical test(s) used AND whether they are one- or two-sided<br>*Only common tests should be described solely by name; describe more complex techniques in the Methods section.*
☐	☒	A description of all covariates tested
☐	☒	A description of any assumptions or corrections, such as tests of normality and adjustment for multiple comparisons
☐	☒	A full description of the statistical parameters including central tendency (e.g. means) or other basic estimates (e.g. regression coefficient) AND variation (e.g. standard deviation) or associated estimates of uncertainty (e.g. confidence intervals)
☐	☒	For null hypothesis testing, the test statistic (e.g. *F*, *t*, *r*) with confidence intervals, effect sizes, degrees of freedom and *P* value noted<br>*Give P values as exact values whenever suitable.*
☒	☐	For Bayesian analysis, information on the choice of priors and Markov chain Monte Carlo settings
☒	☐	For hierarchical and complex designs, identification of the appropriate level for tests and full reporting of outcomes
☐	☒	Estimates of effect sizes (e.g. Cohen's *d*, Pearson's *r*), indicating how they were calculated

Our web collection on statistics for biologists contains articles on many of the points above.

Software and code

Policy information about availability of computer code

Data collection	Seurat v2.3.4, Seurat v3.14, R v3.5.0
Data analysis	scPhere v0.1.0, Python v3.6.8, R v3.5.0, Liger v0.5.0, Harmony v1.0, uwot v0.1.10, phateR 1.0.4, FIt-SNE v1.1.0

For manuscripts utilizing custom algorithms or software that are central to the research but not yet described in published literature, software must be made available to editors/reviewers. We strongly encourage code deposition in a community repository (e.g. GitHub). See the Nature Research guidelines for submitting code & software for further information.

Data

Policy information about availability of data

All manuscripts must include a data availability statement. This statement should provide the following information, where applicable:

- Accession codes, unique identifiers, or web links for publicly available datasets
- A list of figures that have associated raw data
- A description of any restrictions on data availability

We used publicly available datasets in this study (GEO: GSE12695438, GSE11956241, GSE13014839, GSE11158840, GSE13740042, GSE13435543, GSE12695452, GSE10658749; Single Cell Portal: SCP259). To make the results presented in this study reproducible, all processed data are available in the Single Cell Portal (SCP551).

Field-specific reporting

Please select the one below that is the best fit for your research. If you are not sure, read the appropriate sections before making your selection.

Life sciences study design

All studies must disclose on these points even when the disclosure is negative.

Sample size	We analyzed nine datasets in this study. We first applied scPhere to six scRNA-seq datasets from human and mouse, spanning from small (thousands) to very large (hundreds of thousands) of cells from one or multiple tissues, and with a small (two) to very large (dozens) of expected cell types. The "small" datasets were: (1) a blood cell dataset with only 10 erythroid cell profiles and 2,293 CD14+ monocytes; (2) 3,314 human lung cells, (3) 1,378 mouse white adipose tissue stromal cells, and (4) 1,755 human splenic nature killer cells spanning four subtypes. The "large" datasets were: (1) 35,699 retinal ganglion cells in 45 cell subsets; and (2) 599,926 cells spanning 102 subsets across 59 human tissues in the Human Cell Landscape. We next analyzed more than 300,000 cells from human colon mucosa, 5,226 zebrafish cells at 50 epiboly, and 86,024 C. elegans embryonic cells. In total, we analyzed more than 1M cells collected from nine studies from four species, produced by four technologies. Thus, we think our data size is sufficient for our study.
Data exclusions	For the colon mucosa data, we removed cells with high percentage of mitochondrial gene transcripts. Specifically, for epithelial cells, we removed cells with more than 35% of mitochondrial gene transcripts. We also removed Enterocyte Progenitors, TA 1, and Immature Enterocytes 1, as these cells have far fewer UMIs, and there were no clear up-regulated genes in these cell clusters. For fibroblasts, similarly, we removed WNT2B+ Fos-lo 2 cells, and also cells with high percentage of mitochondrial gene transcripts (15%). For the cord blood mononuclear cell dataset, we only used the 2,293 CD14+ monocytes and the first 10 erythroid cells to demonstrate the cell crowding problem.
Replication	Although repeated runs produce slightly different embeddings, the results are reproducible in discerning different cell types
Randomization	The allocation was random. Some datasets have specific structures, e.g., the zebrafish embryonic cells are suitable for trajectory inference because they have hierarchical developmental trajectories.
Blinding	We used publicly available datasets to test our algorithms. To evaluate the results we need to know the 'ground-truth' so blinding is not relevant for our study.

Reporting for specific materials, systems and methods

We require information from authors about some types of materials, experimental systems and methods used in many studies. Here, indicate whether each material, system or method listed is relevant to your study. If you are not sure if a list item applies to your research, read the appropriate section before selecting a response.

Materials & experimental systems

n/a	Involved in the study
☒	☐ Antibodies
☒	☐ Eukaryotic cell lines
☒	☐ Palaeontology
☒	☐ Animals and other organisms
☒	☐ Human research participants
☒	☐ Clinical data

Methods

n/a	Involved in the study
☒	☐ ChIP-seq
☒	☐ Flow cytometry
☒	☐ MRI-based neuroimaging
